# Structure and mechanism of a redesigned multidrug transporter from the Major Facilitator Superfamily

**DOI:** 10.1038/s41598-020-60332-8

**Published:** 2020-03-03

**Authors:** Hsin-Hui Wu, Jindrich Symersky, Min Lu

**Affiliations:** Center for Proteomics and Molecular Therapeutics, Rosalind Franklin University of Medicine and Science, 3333 Green Bay Road, North Chicago, IL 60064 USA

**Keywords:** Biochemistry, Structural biology

## Abstract

The rapid increase of multidrug resistance poses urgent threats to human health. Multidrug transporters prompt multidrug resistance by exporting different therapeutics across cell membranes, often by utilizing the H^+^ electrochemical gradient. MdfA from *Escherichia coli* is a prototypical H^+^ -dependent multidrug transporter belonging to the Major Facilitator Superfamily. Prior studies revealed unusual flexibility in the coupling between multidrug binding and deprotonation in MdfA, but the mechanistic basis for this flexibility was obscure. Here we report the X-ray structures of a MdfA mutant E26T/D34M/A150E, wherein the multidrug-binding and protonation sites were revamped, separately bound to three different substrates at resolutions up to 2.0 Å. To validate the functional relevance of these structures, we conducted mutational and biochemical studies. Our data elucidated intermediate states during antibiotic recognition and suggested structural changes that accompany the substrate-evoked deprotonation of E26T/D34M/A150E. These findings help to explain the mechanistic flexibility in drug/H^+^ coupling observed in MdfA and may inspire therapeutic development to preempt efflux-mediated antimicrobial resistance.

## Introduction

Integral membrane proteins known as multidrug transporters curtail the efficacy of existing and new therapeutics by extruding them across cell membranes^1,2^. Multidrug transporters pose catastrophic and growing threats to humankind as they can confer multidrug resistance to pathogenic microorganisms and human cells, which in turn renders the associated diseases untreatable. It has been estimated that multidrug-resistant infectious diseases alone claim >2 million lives and cost > $170 billion each year^3^. Indeed, our arsenal of new antimicrobials and other therapeutic drugs is being depleted and outstripped by the rapid increase in multidrug resistance^2^. As such, the public-health crisis caused by multidrug resistance cannot be solved unless we understand the underlying mechanisms. Unfortunately, despite recent progress, we still lack a deep and mechanistic understanding of multidrug extrusion^4^.

So far, at least seven families of multidrug transporters have been identified^4^: the ABC (ATP-binding cassette), the AbgT (*p*-aminobenzoyl-glutamate transporter), the DMT (drug/metabolite transporter), the MATE (multidrug and toxic compound extrusion), the MFS (major facilitator superfamily), the PACE (proteobacterial antimicrobial compound efflux), and the RND (resistance-nodulation-division) families^4^. Nearly half of the membrane transporters known to date belong to the ubiquitous ABC and MFS protein families, and the majority of multidrug transporters employ the inwardly directed H^+^ electrochemical gradient to export drugs^1,4^. Among them, the MFS represents the largest family of solute transporters with >1 million sequenced members and consists of >82 subfamilies^5–7^.

Previous studies of MFS transporters such as LacY (lactose permease) and GLUTs (glucose transporters), which are from the oligosaccharide/H^+^ symporter (OHS) and sugar porter (SP) subfamilies, respectively, revealed the shared transporter architecture and provided critical insights into the underlying mechanism^8,9^. Particularly, it was established that the substrate-specific LacY and GLUTs utilize extensive H-bonding networks in recognizing their cognate substrates. Moreover, the coupling between substrate- and H^+^-binding in the H^+^-dependent LacY is highly specific, which depends on the dense and precise networks of H-bonds implicated in substrate- and H^+^-binding. Therefore, the substrate/H^+^ coupling in LacY is intolerant to the alteration of amino acids involved in these H-bonding interactions^10,11^. Apparently, the exact chemical structure of the LacY substrate imposes stringent structural restraints on the substrate/H^+^ coupling mechanism^8^.

By contrast, MdfA from *Escherichia coli*, a polyspecific MFS multidrug transporter in the ubiquitous drug/H^+^ antiporter-1 (DHA1) subfamily, can export physicochemically disparate compounds that lack a common chemical structure^12,13^. Previous studies suggested that MdfA is mechanistically distinct from LacY^14–21^, although they share the same protein fold^22–26^. Notably, the interactions between MdfA and its substrates are often hydrophobic and ionic in nature, direct H-bonds are scanty^22,26^. Moreover, the coupling between drug binding and deprotonation in MdfA is surprisingly flexible, as a functional MdfA variant can be produced by simply rearranging the substrate- and H^+^-binding protein residues^17–19^. This finding suggested that the alteration of drug/H^+^ coupling in MdfA does not require drastic changes in the transporter structure. Such mechanistic plasticity is striking but presumably is shared by other multidrug transporters from the DHA1 subfamily^27,28^, highlighting that these proteins operate through a seemingly non-canonical and yet flexible drug/H^+^ coupling mechanism.

Since drug/H^+^ coupling is the key to multidrug extrusion^12,13^, we aimed to elucidate the molecular underpinnings of the flexible substrate/H^+^ coupling in bacterial multidrug transporters. Toward this goal, we carried out structural and functional studies of a triple mutant of MdfA, E26T/D34M/A150E, in which the substrate binding and protonation sites are rearranged and yet the multidrug transport function is retained^18^. Our results revealed how this redesigned bacterial multidrug transporter interacts with different substrates and suggested how the antiport function of MdfA tolerates the rearrangement of both drug-binding and protonation sites. Furthermore, we observed structural changes that accompany the substrate-induced deprotonation of E26T/D34M/A150E. Collectively, our findings shed new light on the mechanistic and structural basis of polyspecific substrate recognition and drug/H^+^ coupling, which may pave the way for therapeutic development to battle antimicrobial resistance.

## Results

### Overall structure of E26T/D34M/A150E

In order to study the drug/H^+^ coupling mechanism of E26T/D34M/A150E, we set out to determine its substrate-bound structure by using X-ray crystallography. The best diffracting crystals of E26T/D34M/A150E were obtained in the presence of chloramphenicol (Cm), an electroneutral substrate^18^, at pH 8.0. We cracked the phase problem by using a combination of molecular replacement and SAD (single-wavelength anomalous dispersion) phasing (Supplementary Table 1). Although not essential for solving the phase problem, the density-modified SAD phases were useful for discerning potential structural differences among the MdfA variants^22,26^, for objectively identifying the ligand-binding sites, and for improving the quality of structural models.

**Figure 1:**
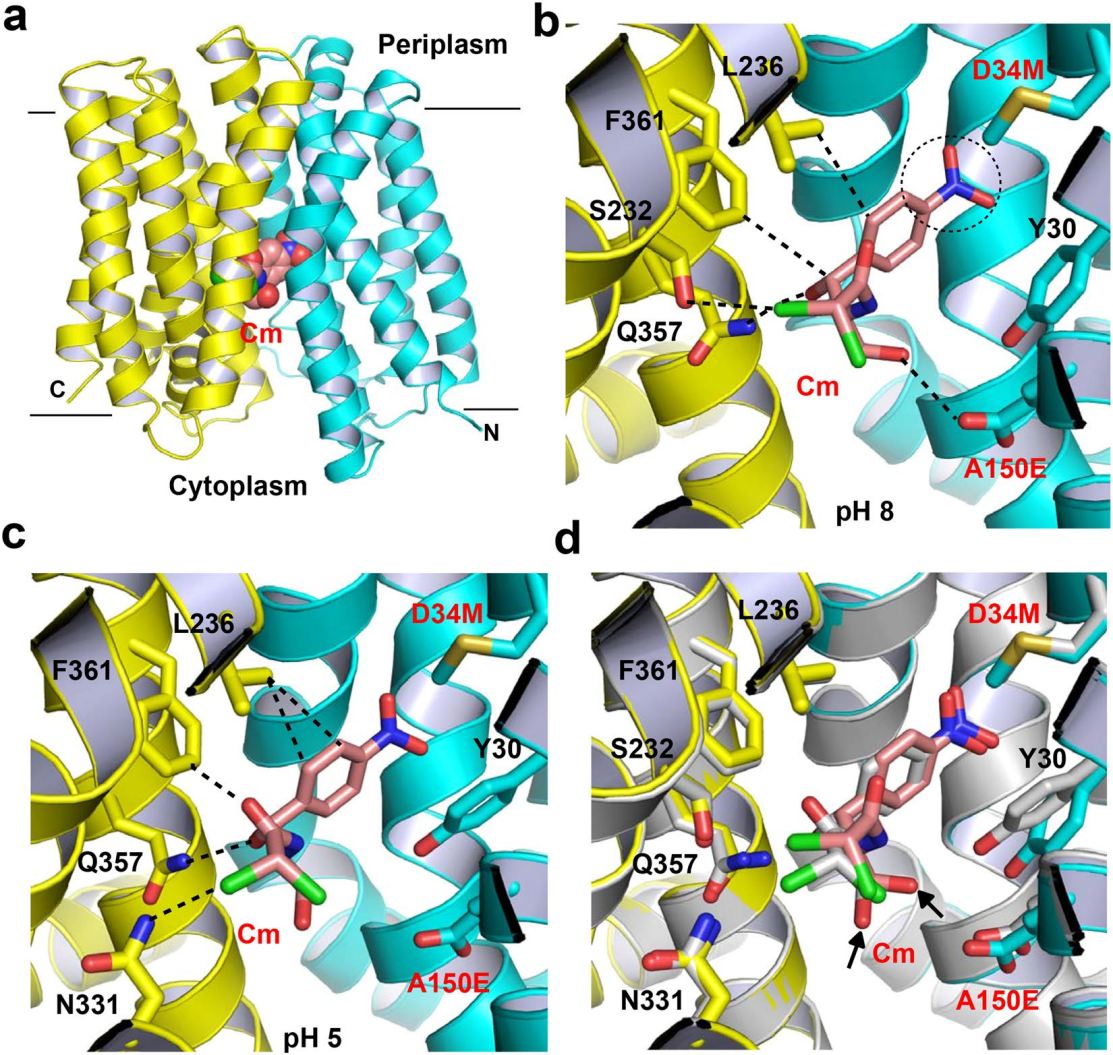
Structures of the chloramphenicol (Cm)-bound E26T/D34M/A150E. (**a**) Structure of E26T/D34M/A150E determined at pH 8.0 as viewed from the membrane. E26T/D34M/A150E is drawn as a ribbon diagram, with the N (14–205) and C (206–400) domains colored cyan and yellow, respectively. The bound Cm molecule is shown as spheres in light pink. (**b**) Close-up view of the Cm-binding site observed at pH 8.0, the bound Cm and the relevant amino acids are shown in stick models and close-range interactions are highlighted as dashed lines. Notably, 3.1, 3.1 and 4.0 Å were used as the distance cutoffs to identify ionic, H-bonding and hydrophobic interactions, respectively. The nitryl group of Cm is highlighted by a black, dotted circle. (**c**) Close-up view of the Cm-binding site identified at pH 5.0, Cm and the relevant amino acids are shown in stick models and close-range interactions are highlighted as dashed lines. (**d**) Overlay of the Cm-bound structure of E26T/D34M/A150E at pH 8.0 (same color scheme as in **a**) and that of E26T/D34M/A150E at pH 5.0 (gray), with the Cm-binding amino acids and Cm drawn as stick models. Black arrows highlight the O4 atoms of the Cm molecules. Notably, as E26T/D34M/A150E transitions from the low (grey) to high (light pink) pH structure, both the nitryl group of Cm and the side-chain of Y30 move so that the nitryl group in Cm can interact with the edge of the aromatic ring from Y30. This figure is prepared with the software PyMOL, version 2.3.2, http://pymol.org.

The resulting structure of E26T/D34M/A150E has been refined to 2.0 Å resolution and revealed a transporter structure that is similar to that of Cm-bound MdfA variant Q131R^22^, with an rms deviation of 1.0 Å for 387 common Cα atoms. Q131R is a fortuitous single mutant that was identified during the crystallographic pursuit of ligand-bound MdfA, which likely represents the wild type transporter when protein-ligand interactions are analyzed^22^. This structural similarity suggested that our E26T/D34M/A150E structure portrays the transporter in its inward-facing conformation and the triple mutation E26T/D34M/A150E did not perturb the protein structure to any significant degree. As the Cm-bound structure of Q131R, E26T/D34M/A150E exhibits the same structural fold as LacY, comprised of twelve membrane-spanning helices that can be divided into two similarly folded domains, the N and C domains (Fig. 1a).

### Capture of two Cm-binding modes in the crystals

At the interface between the N and C domains, the Cm-binding site was identified based on the experimental electron density maps calculated with the density-modified SAD phases (Supplementary Fig. 1). Located approximately halfway across the membrane bilayer, the bound Cm engages in a number of close-range interactions with the transporter (Fig. 1b). Specifically, the O4, O5 and CL1 atoms in Cm form direct H-bonds with the side-chains of A150E (OE1), Q357 (NE2) and S232 (OG), respectively (Supplementary Table 2). Moreover, the bound Cm makes van der Waals interactions with the side chains of L236 and F361. In addition, the nitrobenzene moiety of Cm appears to interact favorably with the side-chain phenol of Y30, since the electronegative nitryl group of Cm is 3.3 Å from the electropositive edge of the aromatic ring in Y30. Notably, the side-chain carboxylate of A150E has a calculated pK_a_ of 6.6 (ref. ^29^), which is similar to the experimentally measured pK_a_ of 6.5 (ref. ^19^). Thus, given the pH (8.0) of the crystal solutions, our Cm-bound structure of E26T/D34M/A150E likely portrays a substrate-bound, deprotonated transporter. Furthermore, the bound Cm is likely to be electroneutral in this co-crystal structure, since its pK_a_ is ~5.5.

Since E26T/D34M/A150E is an H^+^-coupled multidrug transporter^19^, we next asked if it interacts with Cm differently when the experimental pH is lowered below the pK_a_ of A150E. Hence, we determined the Cm-bound structure of E26T/D34M/A150E at pH 5.0 (Supplementary Table 1), which likely represents a substrate-bound, protonated transporter. The low-pH co-crystal structure revealed similar transporter-Cm interactions (Supplementary Fig. 2 and Supplementary Table 2) to those observed at pH 8.0, but with several exceptions. Firstly, at pH 5.0, the side-chain carboxylate of A150 is 4.4 Å away from the bound Cm, which precludes it from making a direct H-bond (Fig. 1c). By contrast, the bound Cm is 3.0 Å from A150 in E26T/D34M/A150E at pH 8.0. Secondly, the CL1 atom of Cm interacts with the side chain of N331 rather than S232 through a direct H-bond. Lastly, the electronegative nitryl group of Cm is positioned atop the aromatic ring of Y30, implying an energetically more unfavorable arrangement of the nitrobenzene moiety and side-chain phenol than that seen at pH 8.0 (ref. ^30^).

These structural differences indicated that the switchover from a Cm-bound, protonated E26T/D34M/A150E (Fig. 1c) to its deprotonated counterpart (Fig. 1b) involves an increase in the number of transporter-substrate interactions, because in the latter, the O4 atom in Cm forms an H-bond with A150E and the nitryl group of Cm interacts favorably with Y30 (Fig. 1d). Notably, both the substrate (especially the dichloromethyl group of Cm) and the transporter (especially the side-chain phenol of Y30) make discernable structural changes as E26T/D34M/A150E transitions from the protonated state to the deprotonated form. As a direct consequence of this structural change, Cm H-bonds directly with A150E. Additionally, at pH 5.0, the bound Cm may be partially protonated. However, the protonation state of Cm is unlikely to impact the transporter-ligand interactions because the side-chain carboxylate of A150E is protonated at pH 5.0, and hence the protonatable atom in Cm (N2), which is 5.9 Å from A150E, is unlikely to interact with E26T/D34M/A150E via long-range electrostatic interactions.

### Functional importance of the Cm-binding sites

**Figure 2:**
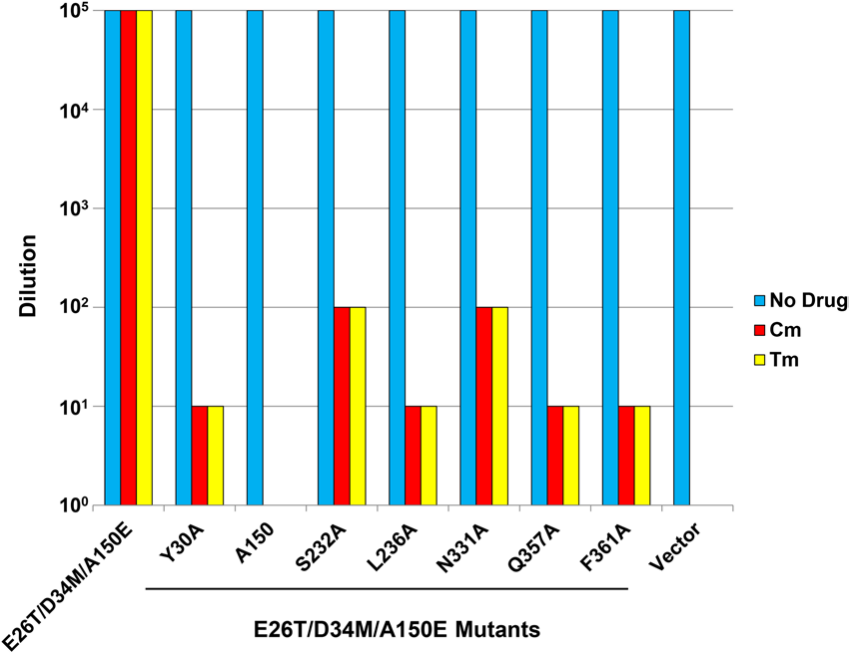
Chloramphenicol (Cm) and thiamphenicol (Tm) resistance assays. Bacteria expressing the E26T/D34M/A150E variants were tested for Cm or Tm resistance in solid media. Five consecutive 10-fold dilutions of bacteria were prepared, and 4 µl of each dilution were plated on plates containing kanamycin, IPTG and 0.5 µg/ml Cm or 2 µg/ml Tm. The ability of bacteria to form single colonies was visualized after overnight incubation. The height of the bars corresponds to the maximal dilution at which bacterial growth was detected. Three different transformants were analyzed for each E26T/D34M/A150E variant.

To probe the functional relevance of the observed protein-Cm interactions, we replaced the Cm-binding amino acids individually with alanine. We found that the expression of these mutants is similar to that of E26T/D34M/A150E (Supplementary Fig. 3). We then tested the function of these single mutants in the drug resistance assay, which qualitatively evaluates the ability of a membrane transporter to confer cellular resistance to cytotoxic compounds via drug efflux (Fig. 2 and Supplementary Fig. 4). Of note, we observed that bacteria expressing the vector were unable to grow in the presence of Cm under the tested conditions, suggesting that the endogenous efflux transporters exerted negligible effects in our drug resistance assay. Moreover, we found that the alanine substitution of Y30, A150E, L236, Q357 or F361 either completely abrogated or severely impaired the ability of E26T/D34M/A150E to confer Cm resistance to *E. coli* (Fig. 2 and Supplementary Fig. 4). The mutation of S232 or N331, however, had less deleterious effect on the transport function. To rule out the possibility that the Y30A, L236A, Q357A, or F361A mutation impaired the transport function by causing misfolding of the transporter, we analyzed these mutants by using analytical size exclusion chromatography^31,32^. We observed that these detergent-purified mutants are all well-folded, similar to E26T/D34M/A150E and E26T/D34M (Supplementary Fig. 5). Our data thus validated the functional relevance of our Cm-bound structures and suggested that Y30, A150E, L236, Q357, and F361 are important for the E26T/D34M/A150E-mediated extrusion of Cm.

Furthermore, our structures revealed that no H-bonding interaction is made between E26T/D34M/A150E and the nitrobenzene moiety of Cm, implying that thiamphenicol (Tm), in which the nitryl group of Cm is replaced by a methyl sulfone group^22^, likely interacts with E26T/D34M/A150E similarly to Cm^18^. Therefore, we studied the function of the Cm-binding-site mutants in the Tm susceptibility assay (Fig. 2 and Supplementary Fig. 4). We observed that the mutation of Y30, A150E, L236, Q357 or F361 severely crippled the ability of E26T/D34M/A150E to confer Tm resistance to *E. coli*. As such, our data indicated that Cm and TM behaved similarly in our drug resistance assay, likely due to similar transporter-ligand interactions. Taken together, our data suggested that the transporter-Cm interactions seen in our structures are functionally important, and that Y30, A150E, L236, Q357 and F361 play pivotal roles in the E26T/D34M/A150E-mediated efflux of Tm or Cm.

**Figure 3:**
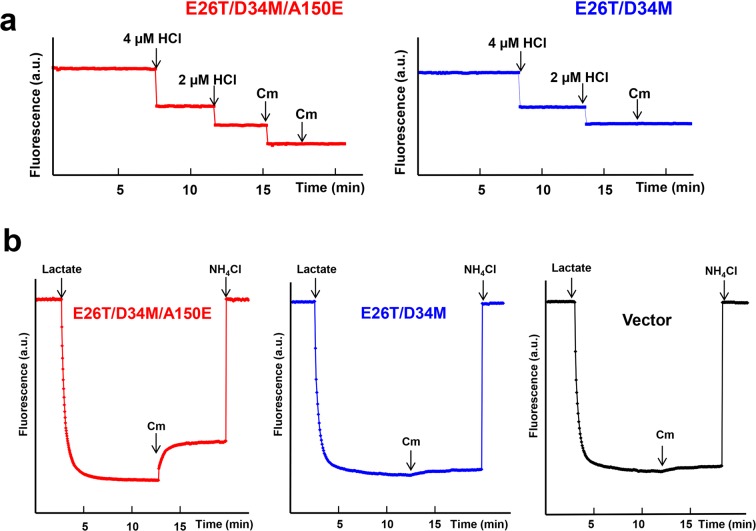
Functional characterization of E26T/D34M/A150E. (**a**) Fluorescence measurement of a solution containing 2 µM E26T/D34M/A150E (red), revealing its ability to release one proton per protein molecule upon chloramphenicol (Cm) binding. As a comparison, the addition of Cm to a solution containing 2 µM E26T/D34M (blue), failed to trigger the release of H^+^. (**b**) Cm/H^+^ antiport observed in the everted membrane vesicles expressing E26T/D34M/A150E (red). H^+^ movement was monitored by measurement of acridine orange fluorescence, which is shown in arbitrary units (a.u.). By contrast, Cm was unable to elicit H^+^ movement in the everted membrane vesicles harboring E26T/D34M (blue) or vector (black). The traces are representative of experiments performed in duplicate using two different preparations of everted membrane vesicles.

Our crystallographic data also implied that Cm triggers the release of H^+^ from the side-chain carboxylate of A150E (Fig. 1b). Concordant with this Cm/H^+^ coupling mechanism, the binding of Cm to the purified E26T/D34M/A150E induced the dissociation of one proton from the transporter, since the magnitude of pH changes elicited by Cm was about the same as the effect of equimolar HCl addition (Fig. 3a). By contrast, the addition of Cm to a solution containing E26T/D34M, which lacks A150E, was unable to evoke the release of any proton from the protein. To corroborate the essential role of A150E in the transporter-mediated export of Cm, we used the inside-out (everted) membrane vesicles to study the Cm/H^+^ antiport^21,26^. We observed that Cm elicited the counter-movement of H^+^ in everted vesicles expressing E26T/D34M/A150E, but not E26T/D34M or vector (Fig. 3b). These data suggested that E26T/D34M/A150E mediated the Cm-induced movement of H^+^ in membrane vesicles, which is unlikely caused by the endogenous efflux transporters in *E. coli*.

Collectively, our results suggested that E26T/D34M/A150E catalyzes the exchange of Cm for a single proton per transport cycle, with A150E serving as the protonation site, and that structural changes in both Cm and the transporter (Fig. 1d) may occur during the substrate-induced deprotonation of A150E. The deprotonation of side-chain carboxylate of A150E, which is likely favored by the alkaline pH in the cytoplasm, may subsequently promote E26T/D34M/A150E to switch from its inward-facing state to the outward-facing conformation^25^.

### Structure of the DXC-bound E26T/D34M/A150E

**Figure 4:**
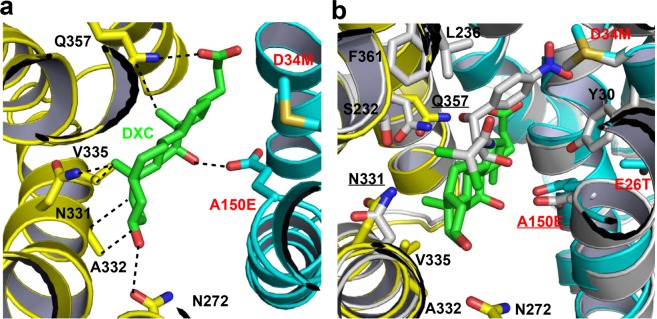
Recognition of deoxycholate (DXC) by E26T/D34M/A150E. (**a**) Close-up view of the DXC-binding site in E26T/D34M/A150E, with the N and C domains colored cyan and yellow, respectively. The bound DXC is drawn in stick models and colored green. The relevant amino acids are shown in stick models and close-range interactions are highlighted by dashed lines. (**b**) Overlay of the DXC-bound structure of E26T/D34M/A150E (same color scheme as in **a**) and that of Cm-bound E26T/D34M/A150E at pH 8.0 (gray), with the substrate-binding amino acids, Cm and DXC drawn as stick models. This figure is prepared with the software PyMOL, version 2.3.2, http://pymol.org.

To examine how E26T/D34M/A150E recognizes electrically different compounds, we determined the 3.0 Å structure of this transporter bound to deoxycholate (DXC) at pH 6.0, by combining molecular replacement and SAD phasing (Supplementary Table 1). The resulting structure is similar to that of Cm-bound E26T/D34M/A150E, with a rms deviation of 0.5 Å over 387 Cα atoms. The experimental maps revealed conspicuous electron density for the bound DXC (Supplementary Fig. 6), anchored at the apex of the cytoplasm-facing, multidrug-binding cavity (Fig. 1a). In particular, the bound DXC engages in van der Waals interactions with the side chains of N331, A232, V335, and Q357 in E26T/D34M/A150E (Supplementary Table 2 and Fig. 4a). Additionally, the side chains of A150E (OE1), N272 (OD1), and Q357 (NE2) form direct H-bonds with the O1, O2 and O3 atoms in DXC, respectively.

Given the pH of the crystallization solution (6.0) and pK_a_ of DXC (6.6), our DXC-bound structure of E26T/D34M/A150E likely portrays the transporter in complex with a protonated DXC. Structure comparison between the DXC- and Cm-bound (deprotonated, pH 8.0) structures of E26T/D34M/A150E further revealed that the bindings sites of the two ligands are partially overlapping, with DXC and Cm both interacting with the side chains of A150E, N331 and Q357 (Fig. 4b). Since a prerequisite for the substrate of any H^+^-coupled multidrug transporter is its ability to trigger the deprotonation of the transporter^26^, and since the H-bonding interaction between DXC and A150E appears adequate to deprotonate the transporter^22,26^, our data suggested DXC as a transportable substrate for E26T/D34M/A150E. Of note, previous studies suggested that DXC can be extruded by MFS transporters including MdfA^26^ and MdtM^33^.

### DXC/H^+^ coupling in E26T/D34M/A150E

**Figure 5:**
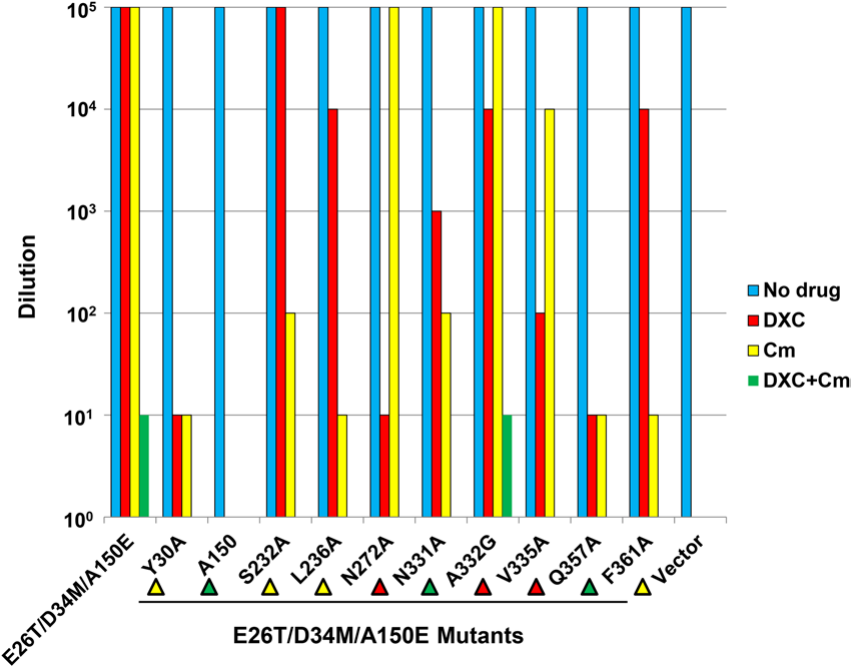
Deoxycholate (DXC) and chloramphenicol (Cm) resistance assays. Bacteria expressing the E26T/D34M/A150E variants were tested for DXC and/or Cm resistance in solid media. Five consecutive 10-fold dilutions of bacteria were prepared, and 4 µl of each dilution were plated on plates containing kanamycin, IPTG, in addition to 300 µg/ml DXC, 0.5 µg/ml Cm, or both 300 µg/ml DXC and 0.5 µg/ml Cm. The ability of bacteria to form single colonies was visualized after overnight incubation. The height of the bars corresponds to the maximal dilution at which bacterial growth was observed. Three different transformants were examined for each E26T/D34M/A150E variant. Yellow, red, and green triangles indicate the mutated amino acids that interact with Cm, DXC, both Cm and DXC, respectively.

To test this hypothesis, we utilized the drug resistance assay to probe whether E26T/D34M/A150E can export DXC (Fig. 5 and Supplementary Fig. 7). Indeed, our data indicated that the expression of E26T/D34M/A150E conferred resistance against DXC to *E. coli*. To further assess the functional importance of the protein-DXC interactions, we mutated the DXC-binding amino acids in E26T/D34M/A150E (Supplementary Fig. 3), and then examined the ability of these mutants to confer resistance against DXC to *E. coli*. We observed that the alanine substitution of A150E completely abolished the ability of E26T/D34M/A150E to render bacteria resistant to DXC, and the mutation of N272 or Q357 to alanine severely impaired the ability of E26T/D34M/A150E to confer cellular resistance against DXC (Fig. 5). Moreover, the mutation of N331 or V335 also had deleterious effect on the transport function, but to a lesser extent. Additionally, detergent-purified mutants N272A and Q357A were all found to be well-folded on the basis of their gel filtration profiles (Supplementary Fig. 5). As such, our data implied that A150E, N272, and Q357 all play crucial roles in the expulsion of DXC by E26T/D34M/A150E.

Our structures also indicated that DXC and Cm compete for the binding of A150E, N331 and Q357 (Fig. 4b), we then tested whether DXC can reduce the ability of E26T/D34M/A150E to confer cellular resistance against Cm (Fig. 5 and Supplementary Fig. 7). We found that the presence of DXC indeed precluded E26T/D34M/A150E from rendering bacteria resistant to Cm. Furthermore, we found the mutation of S232, L236, or F361, which interacts with Cm but not DXC, had little or no adverse effect on the ability of E26T/D34M/A150E to confer resistance against DXC to *E. coli*. Moreover, mutation of N272, A332, or V335, which interacts with DXC but not Cm, had little or no adverse effect on the ability of E26T/D34M/A150E to confer resistance against Cm to *E. coli*. These results are consistent with the observation that the DXC- and Cm-binding sites are only partially overlapping. Of note, the alanine substitution of Y30, which interacts with Cm but not DXC, exerted deleterious effect on the ability of E26T/D34M/A150E to confer cellular resistance against DXC. This finding may be explained by the involvement of Y30 in stabilizing the outward-facing conformation^25^. Additionally, we found that DXC reduced the ability of substrate-binding-site mutants to confer cellular resistance against Cm, regardless of whether the mutated protein residues bind DXC or Cm, thereby implying that DXC inhibits the export of Cm by E26T/D34M/A150E, and vice versa.

**Figure 6:**
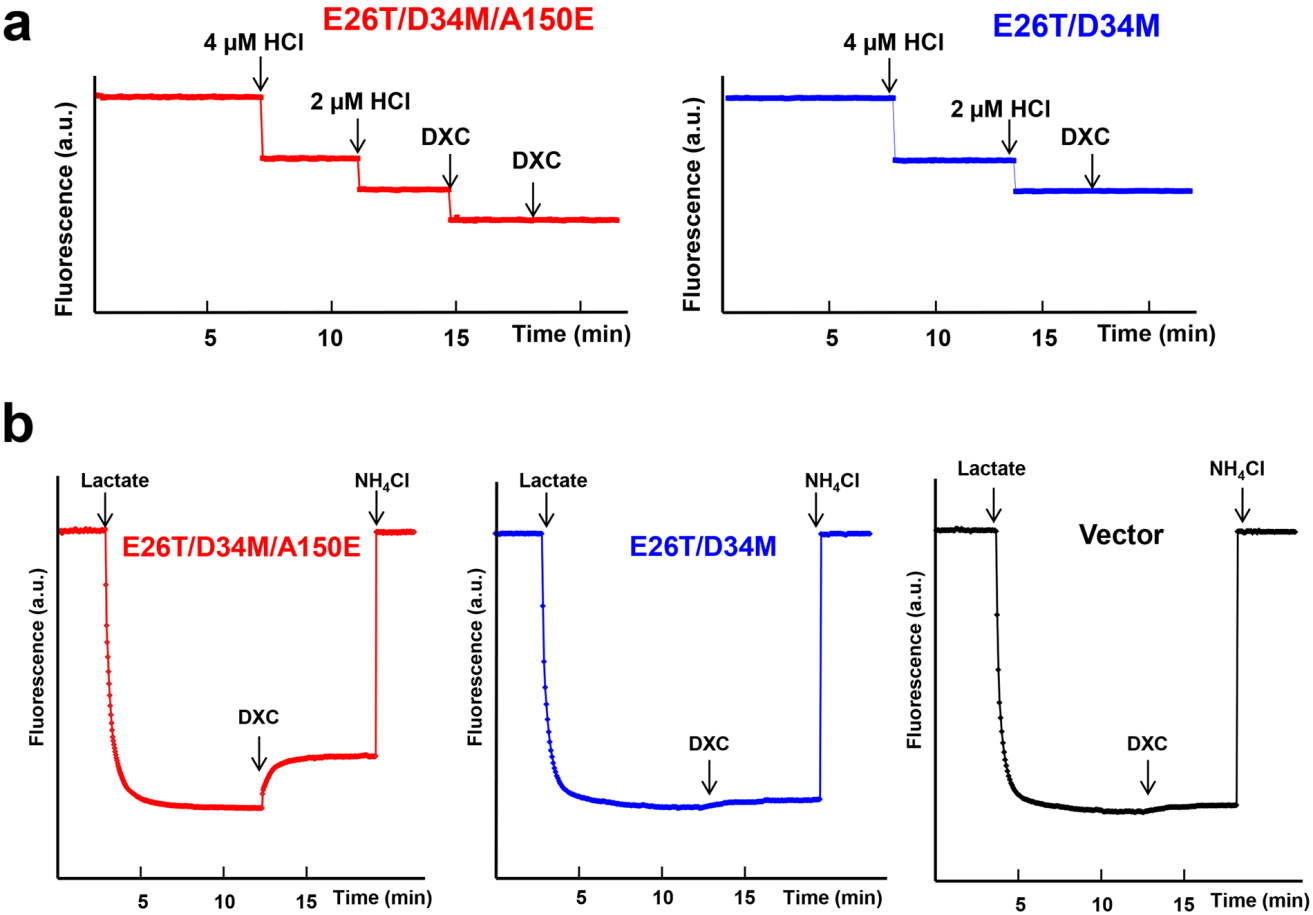
Functional characterization of E26T/D34M/A150E. (**a**) Fluorescence measurement of a solution containing 2 µM E26T/D34M/A150E (red), revealing its ability to release one proton per protein molecule upon deoxycholate (DXC) binding. As a comparison, the addition of DXC to a solution containing 2 µM E26T/D34M (blue), failed to trigger the release of H^+^. (**b**) DXC/H^+^ antiport observed in the everted membrane vesicles expressing E26T/D34M/A150E (red). H^+^ movement was monitored by measurement of acridine orange fluorescence, which is shown in arbitrary units (a.u.). By contrast, DXC failed to elicit H^+^ movement in the everted membrane vesicles harboring E26T/D34M (blue) or vector (black). The traces are representative of experiments performed in duplicate using two different preparations of everted membrane vesicles.

Importantly, our DXC-bound structure of E26T/D34M/A150E also suggested that the transporter catalyzes the exchange of DXC for one proton, with A150E serving as the protonation site. In agreement with this antiport mechanism, we observed that the binding of DXC to the purified E26T/D34M/A150E evoked the stoichiometric release of one proton from the transporter (Fig. 6a). By stark contrast, DXC failed to trigger the release of any proton from the purified E26T/D34M, which lacks A150E. Moreover, DXC was able to induce the H^+^ movement in the inside out membrane vesicles containing E26T/D34M/A150E, but not E26T/D34M, or vector (Fig. 6b). Although DXC carries a negative charge at physiological pH^26^, our data suggested that E26T/D34M/A150E is capable of extruding DXC, most probably via a direct-competition-based coupling mechanism, i.e., DXC and H^+^ compete for the binding of side-chain carboxylate in A150E.

### Structure of the LDAO-bound E26T/D34M/A150E

**Figure 7:**
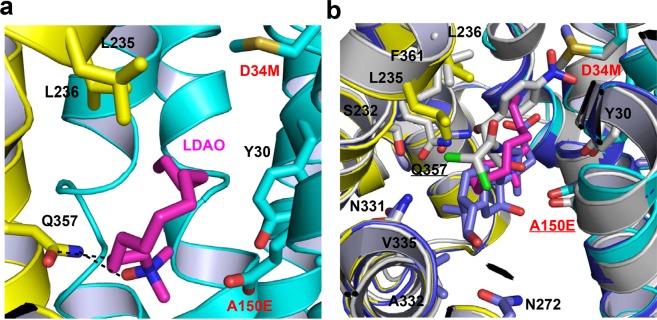
Interactions between E26T/D34M/A150E and LDAO. (**a**) Close-up view of the LDAO-binding site within E26T/D34M/A150E, with the N and C domains colored cyan and yellow, respectively. The bound LDAO molecule (colored magenta) and relevant amino acids are shown in stick models, and the close-range interactions are highlighted by dashed lines. (**b**) Overlay of the LDAO-bound E26T/D34M/A150E and those of the Cm-bound (colored) and DXC-bound (light blue) transporter, with the substrate-binding amino acids and substrates drawn as stick models. This figure is prepared with the software PyMOL, version 2.3.2, http://pymol.org.

Besides anionic DXC and electroneutral Cm, we also determined the 3.0 Å structure of E26T/D34M/A150E bound to zwitterionic LDAO at pH 8.0 by combining molecular replacement and SAD phasing (Supplementary Table 1). The LDAO-bound structure of E26T/D34M/A150E is similar to those of DXC- and Cm-bound complexes, with a pairwise rms deviation of 0.5 Å for 387 equivalent Cα atoms. The experimental electron density maps located the bound LDAO in the cytoplasm-facing cavity (Supplementary Fig. 8), which makes a couple of close-range interactions with E26T/D34M/A150E (Supplementary Table 2). Specifically, the bound LDAO interacts with the side chain of Q357 through both H-bonding and van der Waals interactions (Fig. 7a).

Moreover, the positively charged N1 atom in LDAO is located 4.3 Å away from the negatively charged side-chain carboxylate of A150E (Fig. 7a), likely making long-range electrostatic interactions^26,34–36^, since our structure likely represents a LDAO-bound, deprotonated protein. Besides A150E and Q357, the side chains of Y30, L235 and L236 are ~4.4 Å from the bound LDAO, probably making weak van der Waals interactions. Once the LDAO-bound structure of E26T/D34M/A150E was overlaid onto those of the DXC- and Cm-bound complexes, it became apparent that the LDAO-binding site only partially overlaps with that of DXC or Cm (Fig. 7b). Evidently, the transporter accommodates ligands of varying sizes and charges by utilizing different subsets of protein residues to interact with distinct ligands as well as by adjusting the orientation of side chains of ligand-binding Y30, A150E, L236, N331, and Q357 (Figs. 4b and 7b). This analysis further implied that LDAO competes with Cm or DXC for the binding of E26T/D34M/A150E, since all of the three compounds interact with A150E and Q357.

### LDAO/H^+^ antiport mechanism

**Figure 8:**
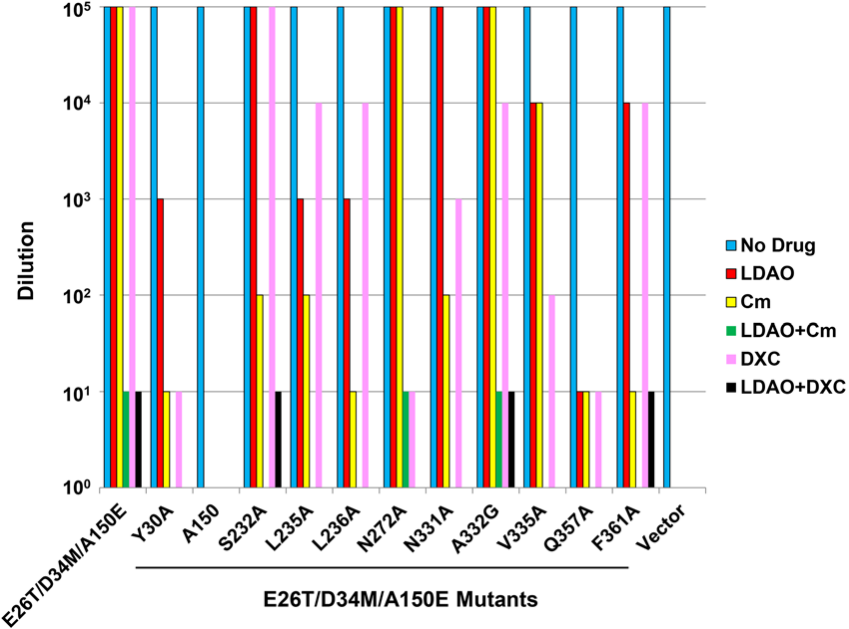
LDAO, chloramphenicol (Cm) and deoxycholate (DXC) resistance assays. Bacteria expressing the E26T/D34M/A150E variants were tested for LDAO, Cm, and/or DXC resistance in solid media. Five consecutive 10-fold dilutions of bacteria were prepared, and 4 µl of each dilution were plated on plates containing kanamycin, IPTG, in addition to 0.01% LDAO, 0.5 µg/ml Cm, 300 µg/ml DXC, or both 0.01% LDAO and 0.5 µg/ml Cm, or both 0.01% LDAO and 200 µg/ml DXC. The ability of bacteria to form single colonies was visualized after overnight incubation. The height of the bars corresponds to the maximal dilution at which bacterial growth was observed. Three different transformants were examined for each E26T/D34M/A150E variant.

We next asked if E26T/D34M/A150E can extrude LDAO. By utilizing the LDAO susceptibility assay, we found that the expression of E26T/D34M/A150E substantially enhanced the resistance of *E. coli* to LDAO, implying that LDAO is also a transportable substrate for E26T/D34M/A150E (Fig. 8 and Supplementary Fig. 9). We then mutated the LDAO-binding amino acids in E26T/D34M/A150E (Supplementary Fig. 3) and tested the ability of these single mutants to confer cellular resistance against LDAO. We found that the alanine substitution of A150E completely abrogated the ability of E26T/D34M/A150E to render *E. coli* resistant against LDAO, whereas the mutation of Q357 to alanine markedly impaired this ability (Fig. 8 and Supplementary Fig. 9). By contrast, the mutation of Y30, L235, or L236 exerted only moderately adverse effects on the transport function. Our data thus implies that A150E and Q357 play pivotal roles in the E26T/D34M/A150E-mediated extrusion of LDAO.

Furthermore, we found that LDAO enhanced the bactericidal effects exerted by Cm or DXC (Fig. 8 and Supplementary Fig. 9), likely by competing for the side chains of both A150E and Q357 (Fig. 7b). The mutation of amino acids that interact with Cm or DXC, but not LDAO, i.e., S232, N272, N331, A232, V335 and F361, however, had little or no deleterious effect on the transport function examined in the LDAO resistance assay (Fig. 8 and Supplementary Fig. 9). Nonetheless, we observed that LDAO decreased the ability of substrate-binding-site mutants to confer cellular resistance against Cm or DXC, regardless of whether the mutated residue binds Cm, DXC, or LDAO, likely because LDAO inhibits the export of Cm or DXC by E26T/D34M/A150E, and vice versa.

**Figure 9:**
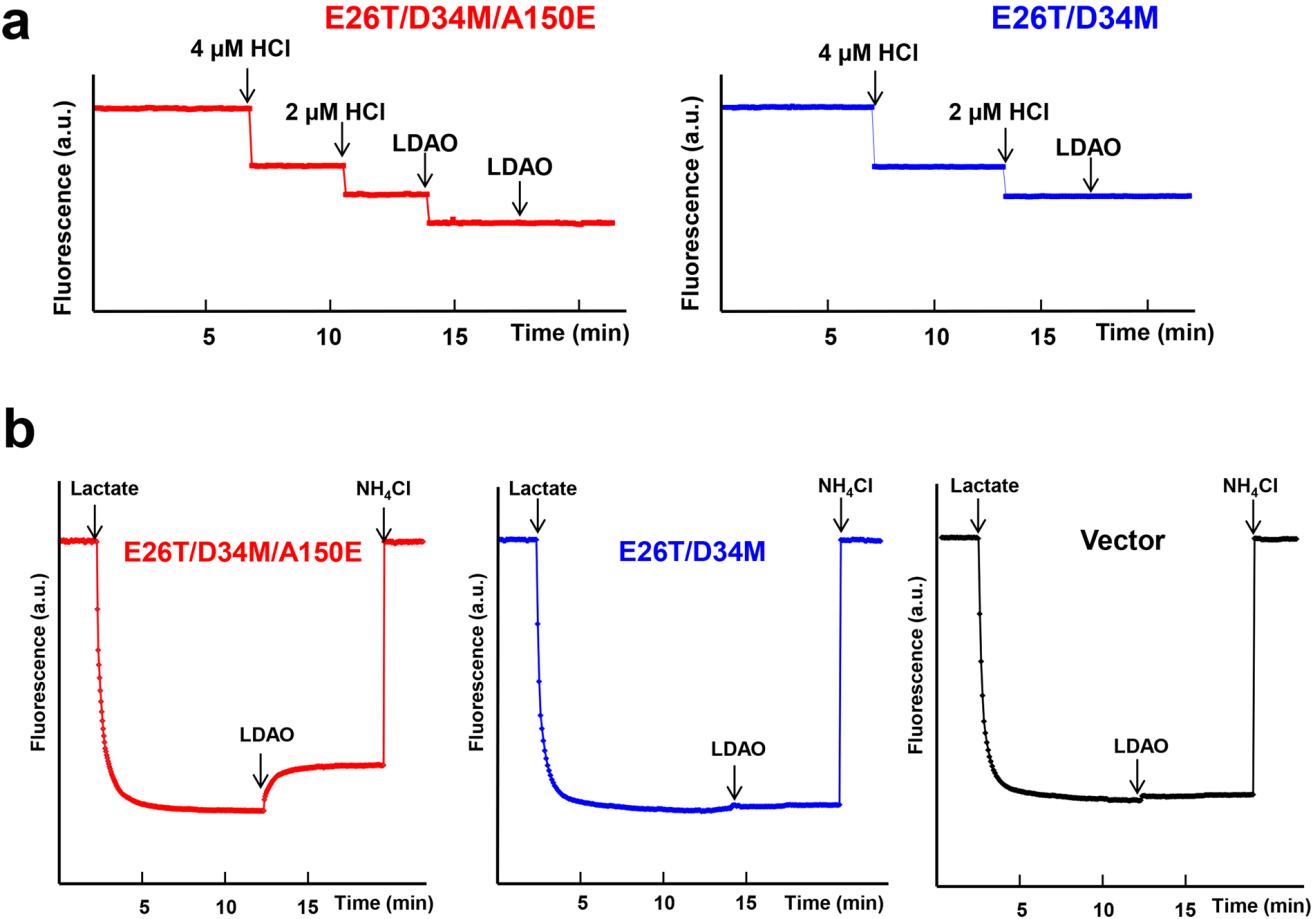
Functional characterization of E26T/D34M/A150E. (**a**) Fluorescence measurement of a solution containing 2 µM E26T/D34M/A150E (red), revealing its ability to release one proton per protein molecule upon LDAO binding. As a comparison, the addition of LDAO to a solution containing 2 µM E26T/D34M (blue), failed to trigger the release of H^+^. (**b**) LDAO/H^+^ antiport observed in the everted membrane vesicles expressing E26T/D34M/A150E (red). H^+^ movement was monitored by measurement of acridine orange fluorescence, which is shown in arbitrary units (a.u.). By contrast, LDAO failed to induce H^+^ movement in the everted membrane vesicles harboring E26T/D34M (blue) or vector (black). The traces are representative of experiments performed in duplicate using two different preparations of everted membrane vesicles.

Our LDAO-bound structure of E26T/D34M/A150E also implied that the transporter catalyzes the exchange of LDAO for one proton, and that LDAO induces the deprotonation of A150E via long-range charge-charge interactions (Fig. 7a). Congruent with these implications, we found that the addition of LDAO to the purified E26T/D34M/A150E resulted in the stoichiometric release of one proton from the transporter (Fig. 9a). By contrast, LDAO was unable to trigger the release of any proton from the purified E26T/D34M, which lacks A150E. Moreover, LDAO induced the H^+^ movement in the inside out membrane vesicles containing E26T/D34M/A150E, but not E26T/D34M, or vector (Fig. 9b). Taken together, our results suggested that A150E acts as the protonation site in E26T/D34M/A150E, and that substrate stimulates the deprotonation of A150E through long-range electrostatic interactions (for zwitterionic LDAO), or via direct H-bonding interactions (for electroneutral Cm and anionic DXC). Furthermore, we contend that monovalent cationic substrate^18^ promotes the deprotonation of E26T/D34M/A150E also through charge-charge interactions, similarly to LDAO.

### Structural comparison with MdfA variant Q131R

**Figure 10:**
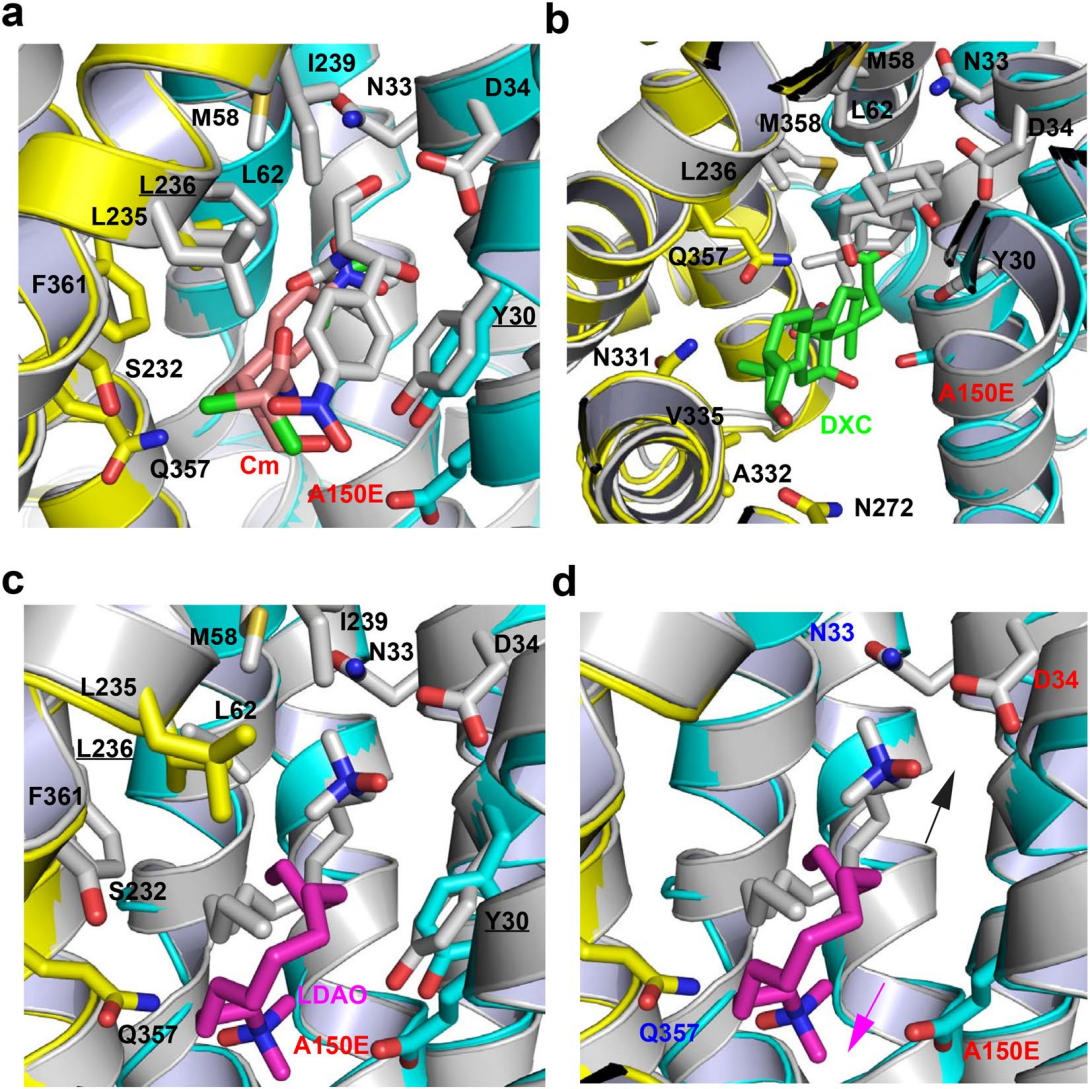
Structural comparison between E26T/D34M/A150E and Q131R. (**a**) Overlay of the Cm-bound structure of E26T/D34M/A150E at pH 8.0 (with the N and C domains colored cyan and yellow, respectively; Cm colored in light pink) and that of MdfA variant Q131R (PDB 4ZOW, colored gray), with the substrate-binding amino acids and Cm drawn as stick models. (**b**) Overlay of the DXC-bound structure of E26T/D34M/A150E (same color scheme as in **a**; DXC colored green) and that of Q131R (PDB 4ZP0, colored gray), with the substrate-binding amino acids and DXC drawn as stick models. (**c**) Overlay of the LDAO-bound structure of E26T/D34M/A150E (same color scheme as in **a**; LDAO colored magenta) and that of Q131R (PDB 4ZP2, colored gray), with the substrate-binding amino acids and LDAO drawn as stick models. (**d**) Overlay of the LDAO-bound structure of E26T/D34M/A150E (same color scheme as in **c**) and that of Q131R (PDB 4ZP2, colored gray) to highlight the protonation sites, with the relevant amino acids and LDAO drawn as stick models. This figure is prepared with the software PyMOL, version 2.3.2, http://pymol.org.

The three substrate-bound E26T/D34M/A150E structures could be superimposed onto those of Q131R^22^ to yield a pairwise rms deviations of ~1.0 Å for 387 common Cα positions. Despite this similarity in protein backbone structures, the three substrates, Cm (Fig. 10a), DXC (Fig. 10b), and LDAO (Fig. 10c), establish interactions with different subsets of protein residues in the two MdfA variants. Of note, the common substrate-binding amino acids are merely Y30 and L236 for Cm or LDAO. For DXC, the substrate-binding amino acids are entirely different between E26T/D34M/A150E and Q131R. Therefore, the triple mutation E26T/D34M/A150E gave rise to drastic changes in the transporter-substrate interactions, further attesting to the remarkable plasticity of the multidrug-binding pocket.

Moreover, overlay of the substrate-bound structures of E26T/D34M/A150E and Q131R revealed distinct binding modes taken by Cm, DXC and LDAO. Notably, the three substrates bound to E26T/D34M/A150E all adopt a different conformer than in the Q131R complexes^22^, in part through a ~180° rotation of each compound along one of its short axes. As such, the distance between the O4 atoms from the transporter-bound Cm molecules exceeds 9.0 Å (Fig. 10a), whereas those between the O2 and N1 atoms from the protein-bound DXC and LDAO molecules exceed 10.0 (Fig. 10b) and 8.5 Å (Fig. 10c), respectively. These spatial separations correlate well with the distance between D34 in Q131R and A150E in E26T/D34M/A150E (10.7 Å measured at the Cα positions), which are located at opposite ends within the multidrug-binding pocket (Fig. 10d).

Somewhat unexpectedly, despite the differences in transporter-substrate interactions, the mechanism underlying the coupling between substrate binding and deprotonation of the MdfA variants remains the same^22,26^. Specifically, electroneutral Cm or anionic DXC seems to trigger the deprotonation of the transporter by forming a direct H-bond with the corresponding side-chain carboxylate, D34 in Q131R or A150E in E26T/D34M/A150E. Zwitterionic LDAO, on the other hand, appears to promote the deprotonation of the transporter through long-range ionic interactions. Apparently, the direct-competition-based drug/H^+^ coupling mechanism is retained by the MdfA variants^26^. These similarities notwithstanding, we observed structural changes that may accompany the Cm-triggered deprotonation of E26T/D34M/A150E (Fig. 1d), which has not been observed in Q131R^22^.

## Discussion

Our findings identified several mechanistic features of the MdfA variants, which may be shared by other multidrug transporters. Firstly, in line with previous studies, our data highlighted the importance of the size of substrate-binding pocket, a crucial determinant of substrate specificity for membrane transporters^37,38^, in achieving polyspecific multidrug recognition. Apparently, the MdfA variants including E26T/D34M/A150E use a large, voluminous substrate-binding pocket (~3000 Å^3^) to interact with different drugs^22,26^. The sheer size of this pocket allows the formation of partially overlapping (Fig. 7b), or completely separate binding sites for substrates^26^, rendering it spatially feasible for the same transporter to bind one or two compounds of very different sizes and shapes. Notably, the large ligand-binding pocket is a structural feature shared not only by multidrug transporters^39–41^, but also by polyspecific transcription factors and enzymes^42,43^, indicating a common strategy in accomplishing polyspecific ligand recognition by proteins.

Secondly, E26T/D34M/A150E and other MdfA variants utilize only a small set of H-bonding interactions to recognize their electroneutral or anionic substrates^22,26^ (Figs. 1b and 4a). Most of the transporter-ligand interactions, however, are mediated through van der Waals and charge-charge interactions (for zwitterionic or cationic drugs)^22,26^, both of which lack stringent requirement for the coordination length or geometry, thereby allowing for substantial flexibility in the transporter-substrate interactions^26,44^. Indeed, previous studies supported the importance of aromatic protein residues in substrate recognition by the MFS transporter MdtM^45^, which is consistent with our observations that several aromatic amino acids are crucial for the multidrug transport mediated by the MdfA variants^26^. Furthermore, the utilization of a small number (typically ≤ 3) of H-bonds in substrate recognition by the MdfA variants starkly contrasts the extensive H-bonding networks (often of ≥8 H-bonds) employed by substrate-specific MFS transporters such as LacY and GLUTs^46,47^, which likely enable structurally and chemically disparate drugs to adopt different orientations within the spacious multidrug-binding pocket (Fig. 7b). Of note, the usage of a small set of H-bonding interactions appears to be yet another common theme in polyspecific ligand recognition by membrane proteins^48^, which probably strikes the right balance between specificity and promiscuity in molecular recognition.

Thirdly, the observation of a Cm-bound, protonated E26T/D34M/A150E, i.e., a fully loaded intermediate state (Fig. 1c), is unexpected, since it was established that the binding of substrate and H^+^ to MdfA is mutually exclusive^19,26^. However, the Cm-bound structure of protonated E26T/D34M/A150E is functionally relevant because the mutations of the substrate-binding protein residues exerted deleterious effects on the transport function (Fig. 2). Notably, previous studies revealed that such a fully loaded state also exists for a Na^+^-coupled multidrug transporter from the MATE family^49^. It thus seems plausible that the drug-induced deprotonation of a multidrug transporter involves more intermediate states than was previously envisioned^50^. On the other hand, since distinct substrates interact with E26T/D34M/A150E differently (Fig. 7b), it remains possible that the extrusion of distinct substrates by the same transporter entails different set of intermediate states, probably with only some substrates (such as Cm) capable of triggering protein structural changes during deprotonation (Fig. 1d).

Fourthly, our results suggested that Cm induces the deprotonation of A150E via direct competition (Figs. 1d and 3a), which in turn confers flexibility to the drug/H^+^ coupling mechanism. Apparently, the relocation of only a single side-chain carboxylate (D34 vs. A150E) is sufficient to alter the multidrug- and H^+^ -binding (Fig. 10d). Our data implied that any side-chain carboxylate within the multidrug-binding pocket, which is usually surrounded by hydrophobic protein residues and in close proximity to a Asn or Gln (N33 in Q131R or Q357 in E26T/D34M/A150E), may meet the requirement for substrate- and H^+^ -binding sites. The hydrophobic and polar residues can enable the transporter to recognize drugs through van der Waals and H-bonding interactions, and the hydrophobic residues can also increase the pK_a_ of the H^+^ -binding side-chain carboxylate^22,26^. Compatible with this flexible design of membrane-embedded substrate- and H^+^ -binding sites, neither D34 in MdfA nor the drug/H^+^ stoichiometry is conserved within the DHA1 subfamily^51^. By contrast, in the substrate-specific LacY, the substrate/ H^+^ coupling relies on the extensive and intricate H-bonding networks, as such, the precise positioning of H^+^- and substrate-binding amino acids is essential for the transport function^11,46^. Therefore, the substrate/H^+^ coupling mechanism of LacY lacks flexibility and the substrate/H^+^ stoichiometry is fixed.

Lastly, our findings suggested that the mechanistic flexibility of MdfA stems from both polyspecific multidrug recognition and direct-competition-based drug/H^+^ coupling. Notably, E26T/D34M/A150E adapts to the rearrangement of multidrug- and H^+^-binding sites by altering the transporter-ligand interactions without compromising the drug/H^+^ antiport function. Thus, antiporters, especially those that operate via a direct-competition-based mechanism, seem mechanistically more flexible than symporters (such as LacY). Indeed, the mutation of a H^+^-binding protein residue in the Na^+^/H^+^ antiporter NapA changed the substrate/H^+^ stoichiometry but failed to disrupt the antiport function^52^. Multidrug antiporters appear to be even more adaptable to the rearrangement of substrate- and/or H^+^-binding sites than the substrate-specific antiporters, because the former are polyspecific in substrate recognition^14,26^. Despite the urgent threats posed by multidrug transporters to human health, the mechanistic insights gleaned from this study provide a simple conceptual framework for understanding the drug/H^+^ coupling mechanism, which may open up new prospects for battling the efflux-mediated antimicrobial resistance.

## Methods

### Protein expression

The gene encoding E26T/D34M/A150E (synthesized by GenScript, NJ) was cloned into a modified pET28b vector, which contains a C-terminal cleavable deca- rather than hexa-histidine tag. The mutations studied in this work were introduced into the gene encoding E26T/D34M/A150E by the QuikChange method (Agilent Technologies), which were subsequently confirmed by DNA sequencing. For membrane transporter overexpression, the *E. coli* BL21 (DE3) Δ*acrAB*Δ*macAB*Δ*yojHI* cells^53^ were transformed with the expression vectors containing the genes encoding the E26T/D34M/A150E variants. The bacteria were cultured in LB media and grew to an OD of 0.6 at 600 nm (OD_600_), at which point they were induced for protein expression with 0.5 mM IPTG at 30 °C for 4 h.

### Protein purification

The cells expressing the E26T/D34M/A150E variants were collected by centrifugation, and then ruptured by multiple passages through a pre-chilled French pressure cell. All the protein purification experiments described in this study were conducted at 4 °C unless specified otherwise. The bacterial membranes were collected by ultracentrifugation, suspended and extracted with 1% (wt/vol) n-dodecyl-β-maltoside (DDM, Anatrace) in 20 mM HEPES-NaOH pH 7.5, 500 mM NaCl, 20% (vol/vol) glycerol and 1.5 mM tris(2-carboxyethyl)phosphine (TCEP). The solubilized membrane sample was centrifuged at 100,000 g for 1 h and the soluble fraction was run over a Ni-NTA column in a buffer containing 20 mM Hepes-NaOH pH 7.5, 100 mM NaCl, 20% glycerol, 0.03% DDM and 1.5 mM TCEP. The protein was eluted from the Ni-NTA column using the same buffer supplemented with 450 mM imidazole. Subsequently, the protein sample was desalted and then incubated with thrombin overnight to remove the deca-histidine tag, in order to avoid its deleterious effects on protein purification or crystallization. The thrombin-cleaved protein sample was desalted and concentrated to a protein concentration of ~20 mg/ml before it was purified by using the size-exclusion chromatography (Superdex 200) in 25 mM Tris-HCl pH 8.0, 150 mM NaCl, 10% glycerol, 0.2% (wt/vol) n-Nonyl-β-Glucoside (NG, Anatrace), 0.025% (wt/vol) n-Dodecyl-N,N-Dimethylamine-N-Oxide (LDAO, Anatrace), 0.05% (wt/vol) deoxycholate (DXC, Sigma) and 1.5 mM TCEP. DDM was used throughout the protein purification for all biochemical studies of the E26T/D34M/A150E variants.

### Protein crystallization and crystal soaking

The purified E26T/D34M/A150E was concentrated to ~10 mg/ml and dialyzed thoroughly against 25 mM Tris-HCl pH 8.0, 150 mM NaCl, 20% (vol/vol) glycerol, 0.2% n-Nonyl-β-Glucoside (NG, Anatrace), 0.025% n-Dodecyl-N,N-Dimethylamine-N-Oxide (LDAO, Anatrace), 0.05% deoxycholate (DXC, Sigma) and 1.5 mM TCEP at 4 °C. All the crystallization experiments were carried out by using the hanging-drop vapor-diffusion method at 24 °C. Ten microliter hanging drops were prepared that included equal volumes of the purified protein samples and a crystallization solution containing 100 mM MES-NaOH, pH 6.0, 150 mM NaCl, 150 mM magnesium chloride, 20 mM praseodymium acetate or Pr(OAc)_3_, 30–40% (wt/vol) PEG400. It generally took between two and four weeks for the E26T/D34M/A150E crystals to reach full size, which grew as parallelogram prisms. These crystals yielded the structure of the DXC-bound complex. To soak chloramphenicol (Cm, Sigma) or LDAO into E26T/D34M/A150E, the protein crystals were incubated in a solution containing 200 mM NaOAc pH 5.0, or 200 mM Tris-HCl, pH 8.0, 150 mM NaCl, 150 mM magnesium chloride, 40 mM Pr(OAc)_3_, 40% (wt/vol) PEG400, 0.2% NG, 30 mM Cm (pH 5.0 or 8.0) or 0.05% LDAO (pH 8.0) at 24 °C for 72 h.

### Structure determination and refinement

The E26T/D34M/A150E crystals were plunged into liquid nitrogen prior to data collection. X-ray diffraction data were collected from the frozen crystals at the beam-lines 23-ID and 22-ID at Argonne National Laboratory. The X-ray data were analyzed by using the program suite HKL2000 (ref. ^54^) and the CCP4 package^55^ unless stated otherwise. All the four structures were solved by combining molecular replacement and SAD phasing. In particular, the Q131R model (PDB 4ZOW)^22^ was located within the unit cell by using the program PHASER^56^. Subsequently, the praseodymium binding sites were determined by conducting difference Fourier analysis and the SAD phases were derived by using the program SHARP^57^. The electron density maps were calculated by using solvent flattening, histogram matching, cross-crystal averaging and phase extension. Model building was performed by using the program O^58^. The structure refinement was conducted by using the program REFMAC with the SAD experimental phases as restraints^59,60^. The resolution cutoffs were chosen on the basis of several criteria, which included the CC_1/2_ (>0.20), data completeness (>50%), and more importantly, whether the inclusion of the X-ray data to the tested resolution limits improved the quality of the structural models and resulting electron density maps^61,62^.

### Western blot analysis

Each sample for western blot analysis was obtained from the bacterial membranes isolated from 80 µg *E. coli* BL21 (DE3) Δ*acrAB*Δ*macAB*Δ*yojHI* cells that expressed the corresponding E26T/D34M/A150E variants. For the western blot, the antibody against the His-tag (Qiagen #34460) were diluted 2500 fold before it was mixed with the transfer membranes. Based on the western blot analysis, it was found that the membrane expression levels of E26T/D34M/A150E variants were not affected by the mutations examined in this study. Thus, the comparison of the transport function of E26T/D34M/A150E mutants in the drug resistance assay is justified.

### Drug resistance assay

The BL21 (DE3) ∆*acrAB*∆*macAB*∆*yojHI* cells expressing the E26T/D34M/A150E variants were cultured at 37 °C in LB media supplemented with 50 µg/ml kanamycin to an OD_600_ of 0.9. Subsequently, five consecutive 10-fold dilutions of cells (10^–1^(–10)^–5^) were performed, and 4 µl of each dilution was pipetted onto the LB plates (2% agar) supplemented with kanamycin (60 µg/ml), IPTG (0.1–0.2 mM) and the tested cytotoxic compounds at various concentrations. For control experiments, the LB plates supplemented with kanamycin (60 µg/ml) and IPTG (0.1–0.2 mM) were used. The ability of the bacteria to form single colonies after 12 h at 30 °C were documented. For each E26T/D34M/A150E variant, three separate transformations were performed.

### Fluorescence measurement of proton-release

This assay was conduct as previously performed^19,21^. Briefly, the DDM-purified E26T/D34M/A150E variants were dialyzed thoroughly against solutions containing 20% (vol/vol) glycerol, 150 mM NaCl and 0.01% (wt/vol) DDM. For each measurement, the protein concentration was adjusted to 2 µM with the same buffer supplemented with 2 µM fluoresceine, a pH-sensitive fluorophore for quantitative measurement of solution acidity^26,63^. The fluorescence measurement was made by using an Olis SLM-8000 spectrofluorometer with excitation and emission wavelengths of 494 nm and 521 nm, respectively. Cm, DXC or LDAO was added at the indicated times to reach a final concentration of 200 µM, 2 mM, and 150 µM, respectively. During measurement the samples (2 ml) were stirred continuously and the temperature was maintained at 10 °C by using a cooling water bath. The same experiments were conducted three times, which yielded a stoichiometry of 1.0 ± 0.1 H^+^ per E26T/D34M/A150E upon the binding of Cm, DXC or LDAO.

### Drug-proton antiport assay

This assay was carried out as previously performed^19,21^. The inside-out (everted) membrane vesicles were prepared from the BL21 (DE3) ∆*acrAB*∆*macAB*∆*yojHI* cells expressing the E26T/D34M/A150E variants^63,64^. In brief, the bacterial cells were cultured in LB media to an OD_600_ of 0.6 and then induced for the protein production with 0.5 mM IPTG at 30 °C for 3 h. Subsequently, the bacterial cells were harvested by centrifugation and washed thoroughly with buffer containing 20 mM Tris-HCl, pH 7.5, 5 mM MgCl_2_, 0.5 mM DTT and 0.25 M sucrose. The cells were ruptured by using a pre-chilled French press and the cell lysate was centrifuged at 10,000 g for 60 min at 4 °C. The bacterial membrane vesicles were collected by centrifugation at 100,000 g for 60 min at 4 °C. To ensure that everted membrane vesicles in similar amounts was used for each measurement, the membrane vesicles that corresponded to 100 µg proteins were pipetted into 2 ml of pre-warmed (30°C) buffer containing 10 mM Tris-HCl, pH 7.0, 5 mM MgCl_2_ and 1 µM acridine orange^26,63,64^. The fluorescence was measured with an excitation wavelength of 492 nm and emission wavelength of 525 nm by using an Olis SLM-8000 spectrofluorometer, and the samples were continuously stirred during the measurement. Before the substrates were introduced, 2 mM lactate was used to energize the membrane and thereby quenched acridine orange fluorescence. For some samples, fluorescence dequenching was observed upon the addition of 100 µM Cm, 250 µM DXC, or 10 µM LDAO, which could be attributed to the extrusion of H^+^ by the antiporters that can translocate the drugs into the membrane vesicles. 5 mM NH_4_Cl was used to dissipate the transmembrane H^+^ gradient. The fluorescence quenching and dequenching signals induced by lactate and NH_4_Cl addition were used to further ensure that similar amounts of everted vesicles of comparable quality were used for each measurement.

## Supplementary information

Supplementary Information.

## Data Availability

Coordinates and structure factors are deposited in the Protein Data Bank under accession codes 6VRZ, 6VS0, 6VS1 and 6VS2. All relevant data supporting the findings of this study are available from the authors upon reasonable request.
