# Supplementary material for: Structure and mechanism of a redesigned multidrug transporter from the Major Facilitator Superfamily
Source: Sci Rep. 2020 Mar 3;10:3949. doi: 10.1038/s41598-020-60332-8 (PMC7054563; doi:10.1038/s41598-020-60332-8)
Supplement: Supplementary file 1 — Supplementary Information. [file 41598_2020_60332_MOESM1_ESM.pdf]

Supplementary Information

Title: Structure and mechanism of a redesigned multidrug transporter from the Major Facilitator Superfamily.

Authors: Hsin-Hui Wu, Jindrich Symersky & Min Lu*

Affiliations: Department of Biochemistry and Molecular Biology, Rosalind Franklin University of Medicine and Science, 3333 Green Bay Road, North Chicago, IL 60064, USA.

*Correspondence should be addressed to M.L.(min.lu@rosalindfranklin.edu)

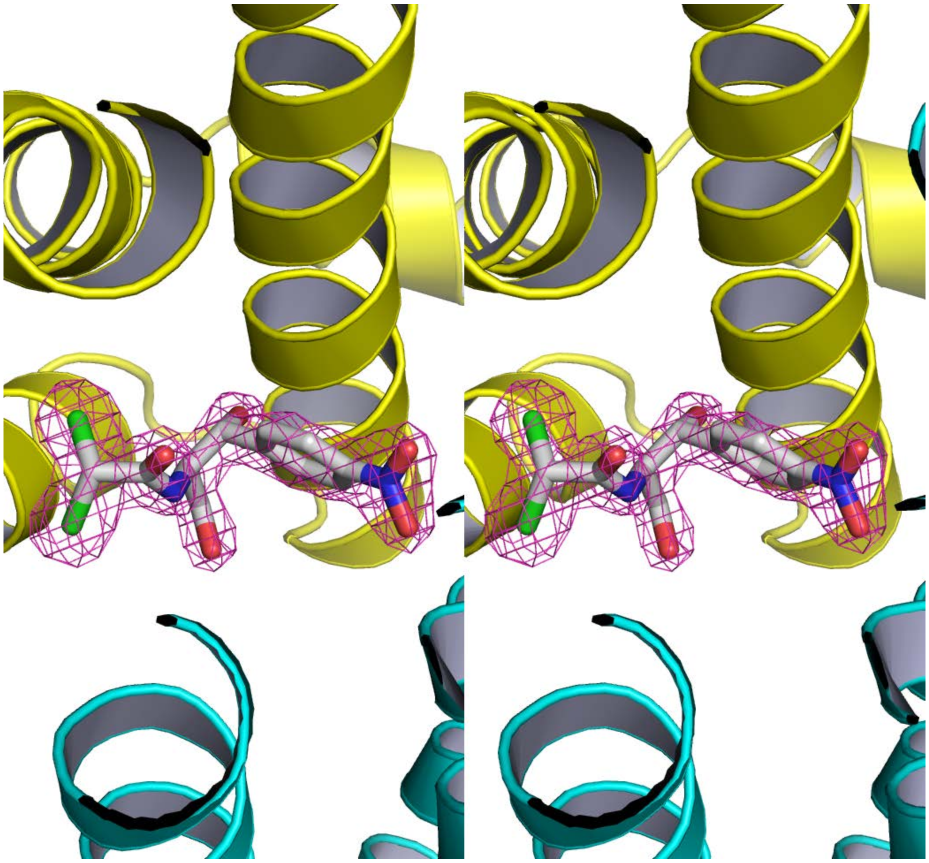

Supplementary Figure 1. Stereo view of the bound chloramphenicol molecule in E26T/D34M/A150E at pH 8.0.

The experimental electron density map (red mesh, 1.5σ) was calculated to 2.0 Å resolution by using the density-modified SAD phases and overlaid onto the final model. Density modification included solvent flattening, histogram matching, cross-crystal averaging and phase extension. E26T/D34M/A150E is shown in ribbon representation and the chloramphenicol molecule is drawn as sticks. The N and C domains of E26T/D34M/A150E are colored cyan and yellow respectively. Chloramphenicol is colored grey. This figure is prepared with the software PyMOL, version 2.3.2, http://pymol.org.

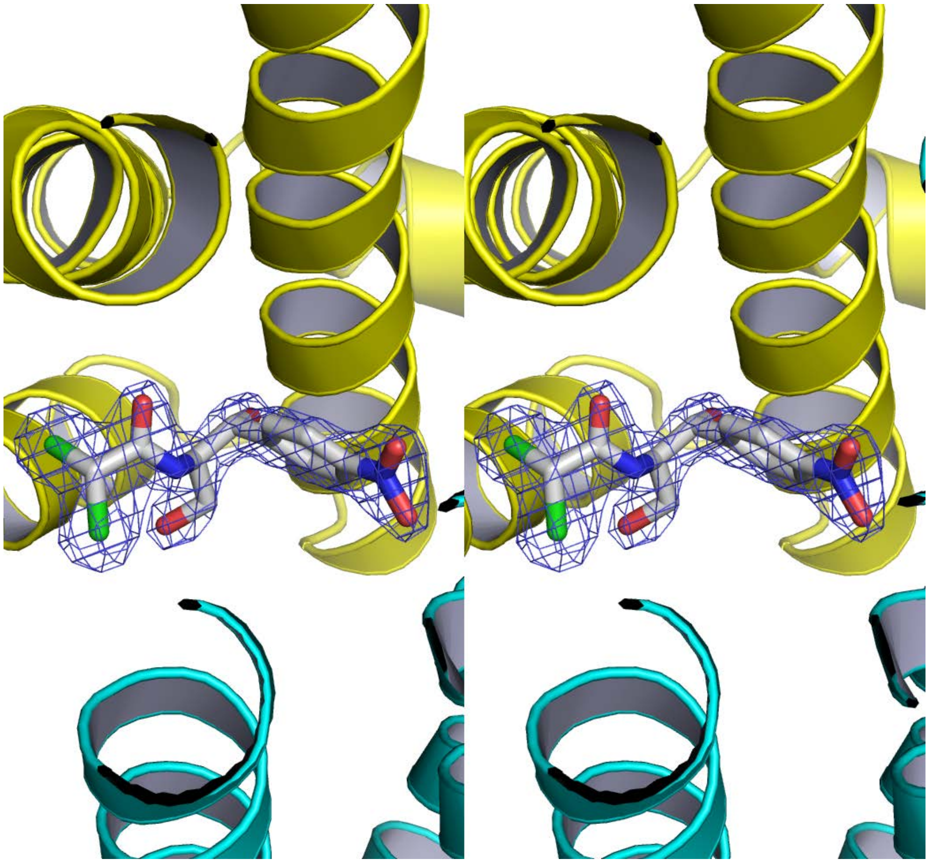

Supplementary Figure 2. Stereo view of the bound chloramphenicol molecule in E26T/D34M/A150E at pH 5.0.

The experimental electron density map (blue mesh, 1.5σ) was calculated to 2.1 Å resolution by using the density-modified SAD phases and overlaid onto the final model. Density modification included solvent flattening, histogram matching, cross-crystal averaging and phase extension. E26T/D34M/A150E is shown in ribbon representation and the chloramphenicol molecule is drawn as sticks. The N and C domains of E26T/D34M/A150E are colored cyan and yellow respectively. Chloramphenicol is colored grey. This figure is prepared with the software PyMOL, version 2.3.2, http://pymol.org.

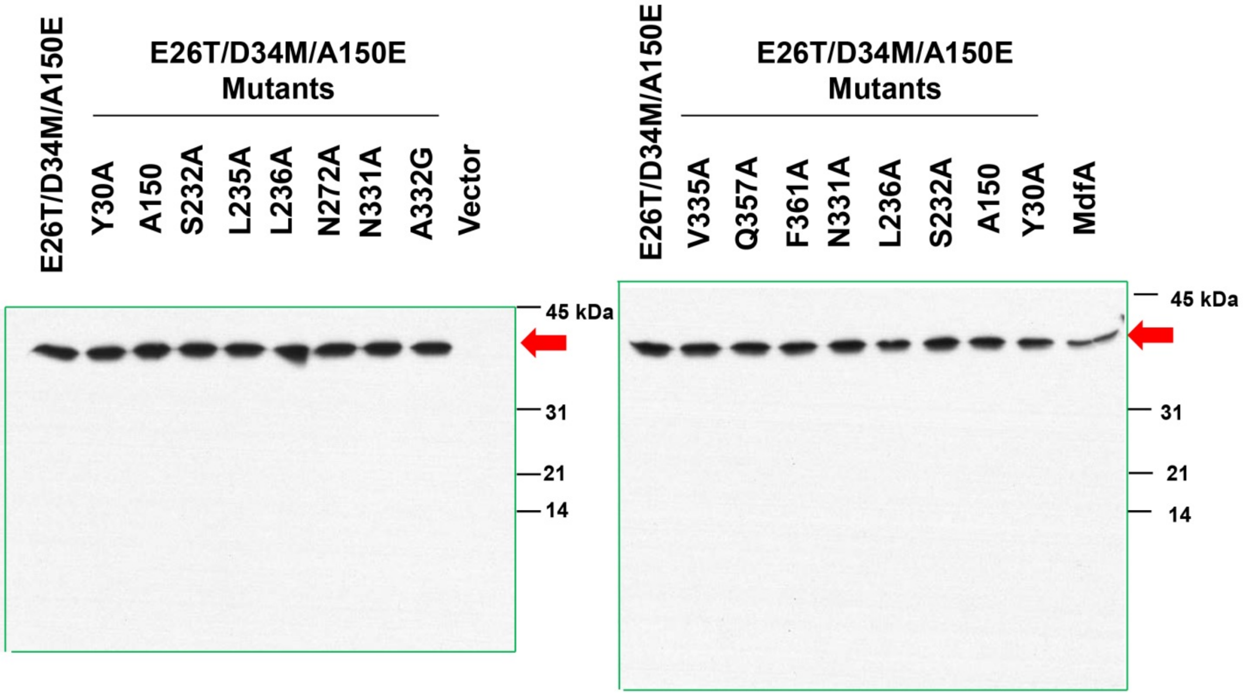

Supplementary Figure 3. Western blot analysis of E26T/D34M/A150E variants.

Western blot analysis of E26T/D34M/A150E variants in membrane preparations, which was performed by using an antibody against the His-tag. This analysis suggested that these proteins were expressed at similar levels. Immunoblots for full-length gels are surrounded by a green line to indicate the borders of the blot. Positions of molecular weight markers are indicated and the bands that correspond to the E26T/D34M/A150E variants are highlighted by red arrows.

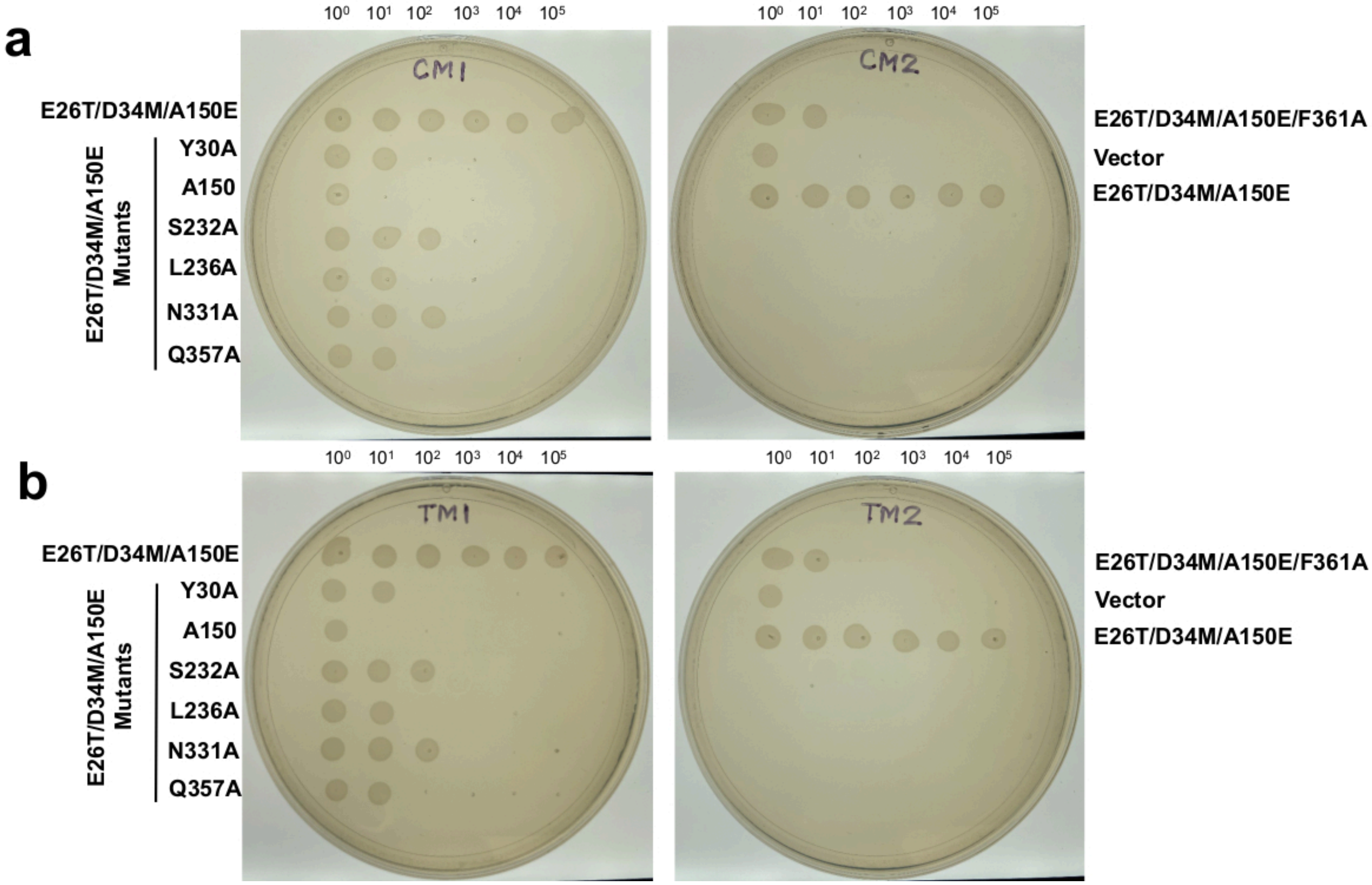

Supplementary Figure 4. Functional characterization of E26T/D34M/A150E variants.

Growth of *E. coli* expressing E26T/D34M/A150E variants on solid media supplemented with kanamycin, IPTG, and chloramphenicol (**a**) or thiamphenicol (**b**). Five consecutive 10-fold dilutions of bacteria were plated from left to right and incubated overnight. Representative results are shown here in the full-length images of LB-agar plates. Of note, bacterial growth on the control LB-agar plates supplemented with kanamycin and IPTG, i.e., in the absence of cytotoxic drugs, was the same for all the E26T/D34M/A150E variants.

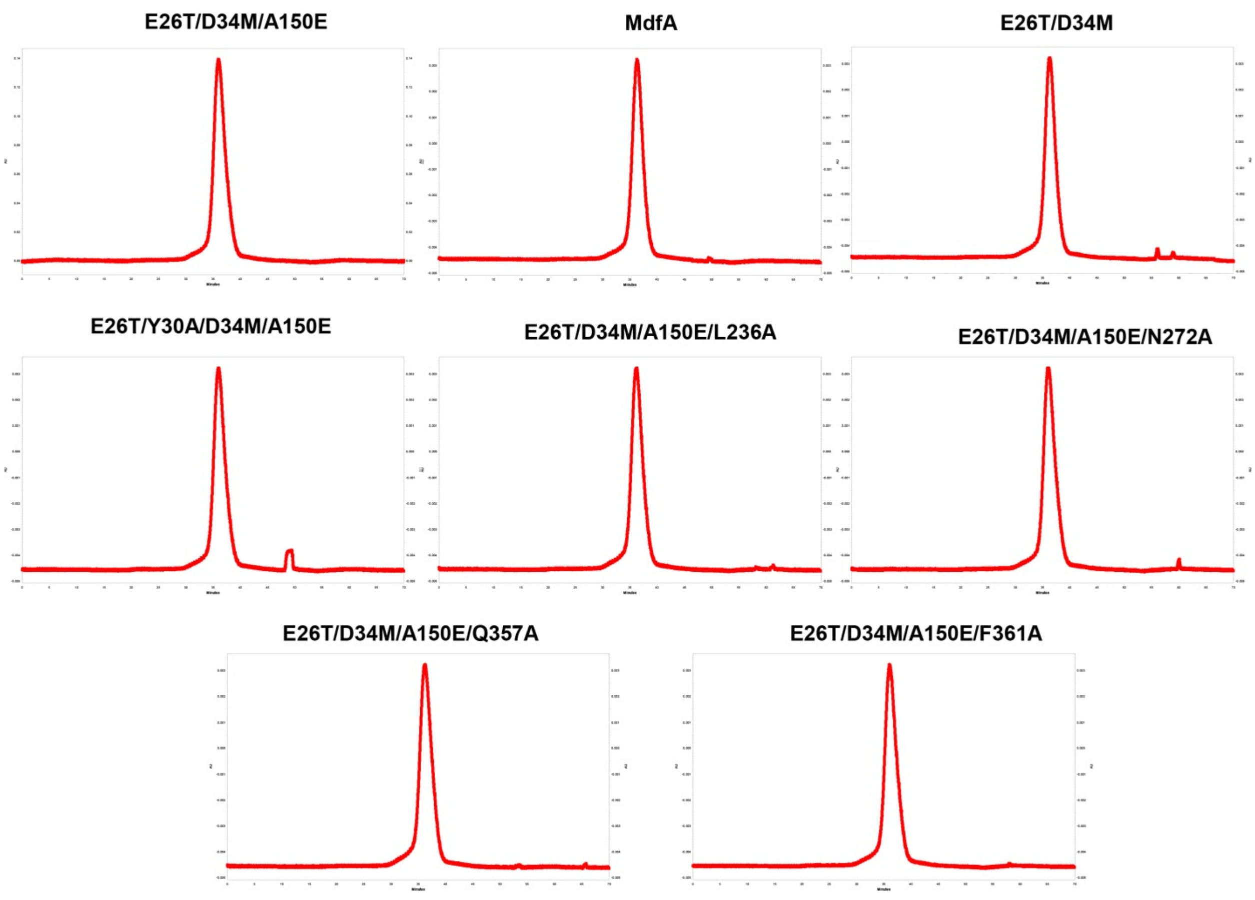

Supplementary Figure 5. Gel filtration chromatograms of E26T/D34M/A150E variants.

For each variant, ~0.2 gram of bacterial membrane was used for purification using the detergent DDM. In each chromatogram, the UV absorption at 280 nm (0-0.16) was plotted against the elution time (0-70 min). The detergent-purified E26T/D34M/A150E variants eluted at ~14ml as sharp, symmetrical peaks, indicating that these proteins are well-folded. For each variant, ~200 µg of protein was loaded onto a Superdex 200 column (~24 ml) pre-equilibrated in 20 mM Tris-HCl pH 8.0, 100 mM NaCl, 10% glycerol, 0.02% DDM and 0.5 mM TCEP, with a flow rate of 0.4 ml/min. All experiments were conducted at 4°C. These size exclusion chromatography results are consistent with our Western blot analysis (Supplementary Fig. 3), suggesting that the tested E26T/D34M/A150E variants were expressed at similar levels in *E. coli*.

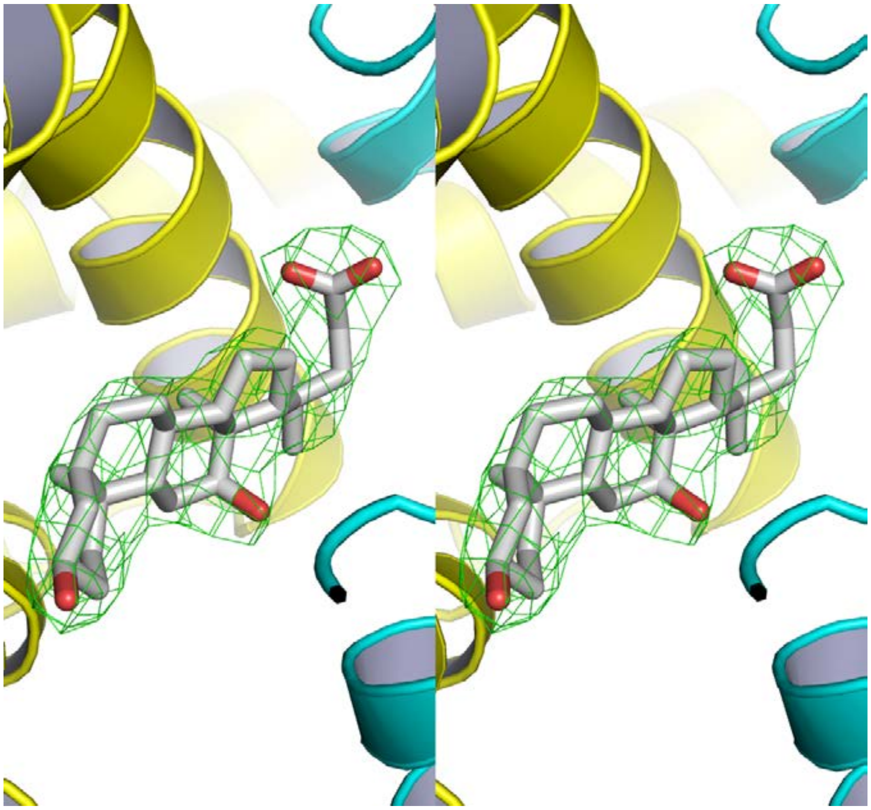

Supplementary Figure 6. Stereo view of the bound deoxycholate molecule in E26T/D34M/A150E.

The experimental electron density map (green mesh, 1.5σ) was calculated to 3.0 Å resolution by using the density-modified SAD phases and overlaid onto the final model. Density modification included solvent flattening, histogram matching, cross-crystal averaging and phase extension. E26T/D34M/A150E is shown in ribbon representation and the deoxycholate molecule is drawn as sticks. The N and C domains of E26T/D34M/A150E are colored cyan and yellow respectively. Deoxycholate is colored grey. This figure is prepared with the software PyMOL, version 2.3.2, http://pymol.org.

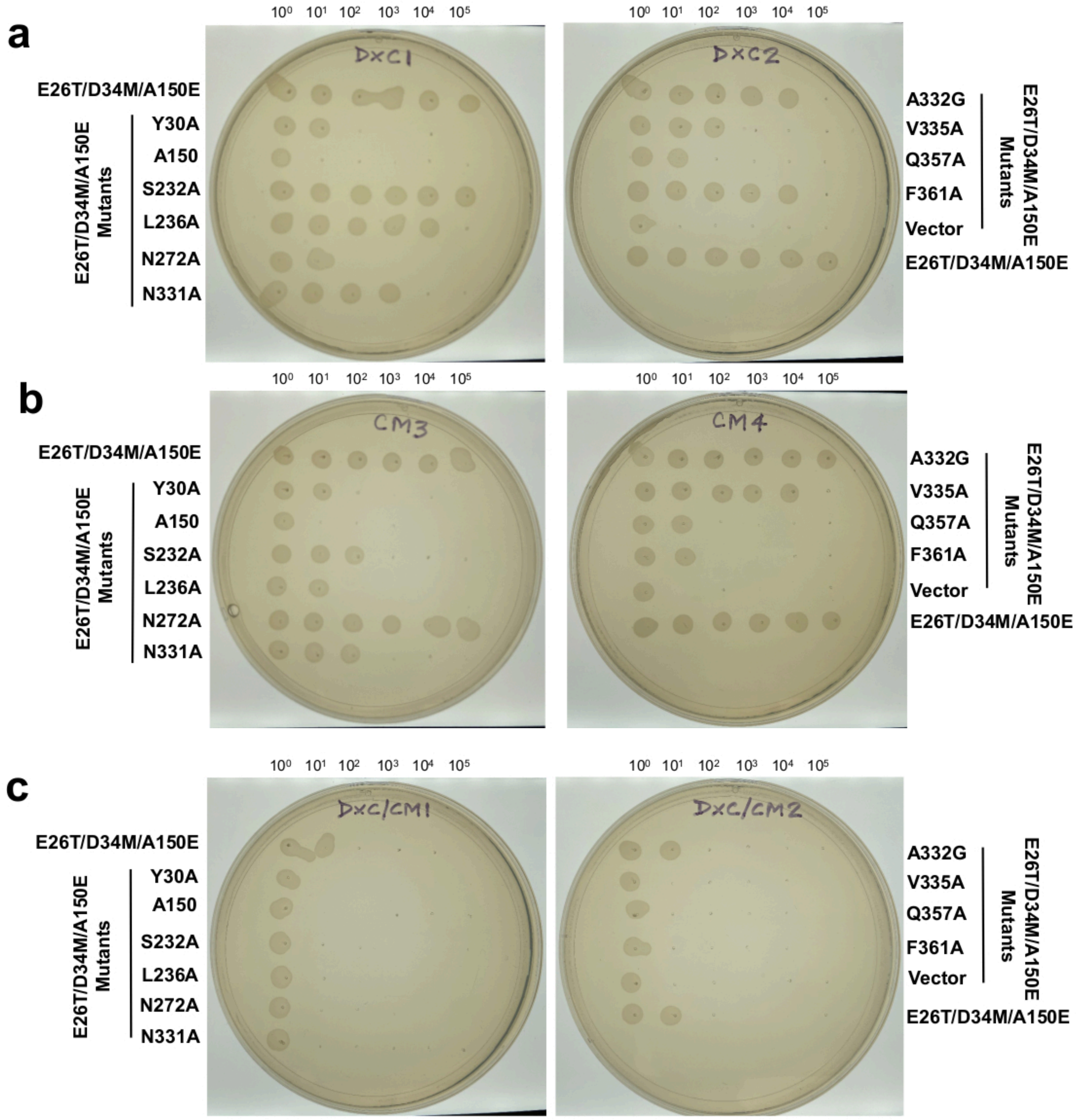
a
b
c
10^0 10^1 10^2 10^3 10^4 10^5
DxC1
DxC2
CM3
CM4
DxC/CM1
DxC/CM2
E26T/D34M/A150E
E26T/D34M/A150E Mutants
Y30A
A150
S232A
L236A
N272A
N331A
A332G
V335A
Q357A
F361A
Vector

Supplementary Figure 7. Functional characterization of E26T/D34M/A150E variants.

Growth of *E. coli* expressing E26T/D34M/A150E variants on solid media supplemented with kanamycin, IPTG, and deoxycholate (**a**), chloramphenicol (**b**) or both deoxycholate and chloramphenicol (**c**). Five consecutive 10-fold dilutions of bacteria were plated from left to right and incubated overnight. Representative results are shown here in the full-length images of LB-agar plates. Of note, bacterial growth on the control LB-agar plates supplemented with kanamycin and IPTG, i.e., in the absence of cytotoxic drugs, was the same for all the E26T/D34M/A150E variants.

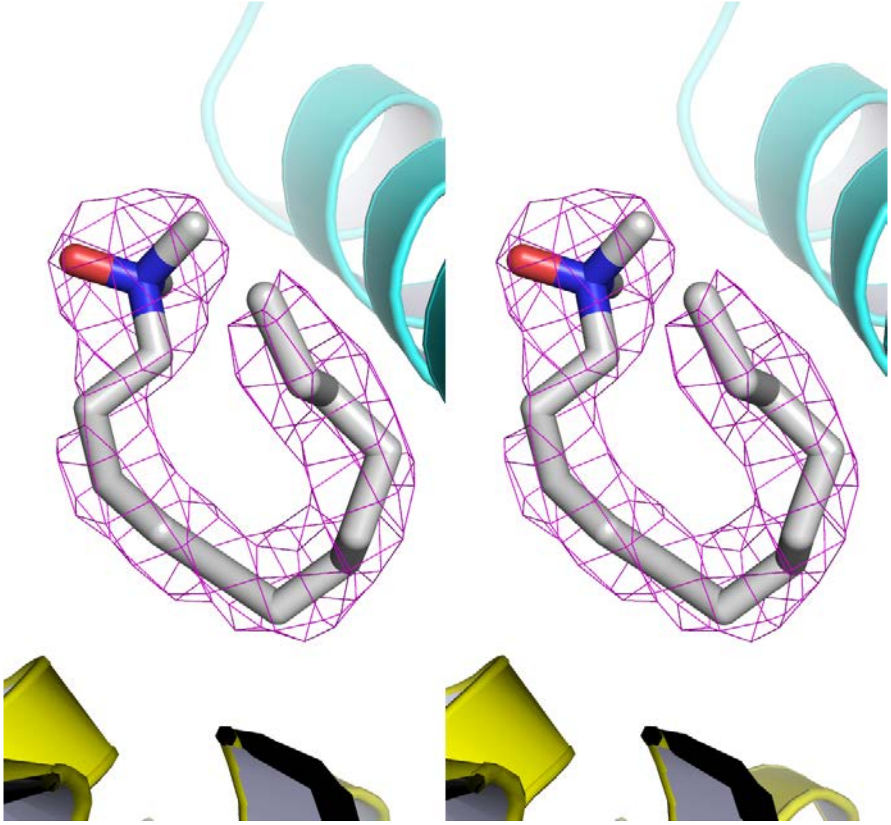

Supplementary Figure 8. Stereo view of the bound LDAO molecule in E26T/D34M/A150E. The experimental electron density map (magenta mesh, 1.5σ) was calculated to 3.0 Å resolution by using the density-modified SAD phases and overlaid onto the final model. Density modification included solvent flattening, histogram matching, cross-crystal averaging and phase extension. E26T/D34M/A150E is shown in ribbon representation and the LDAO molecule is drawn as sticks. The N and C domains of E26T/D34M/A150E are colored cyan and yellow respectively. LDAO is colored grey. This figure is prepared with the software PyMOL, version 2.3.2, http://pymol.org.

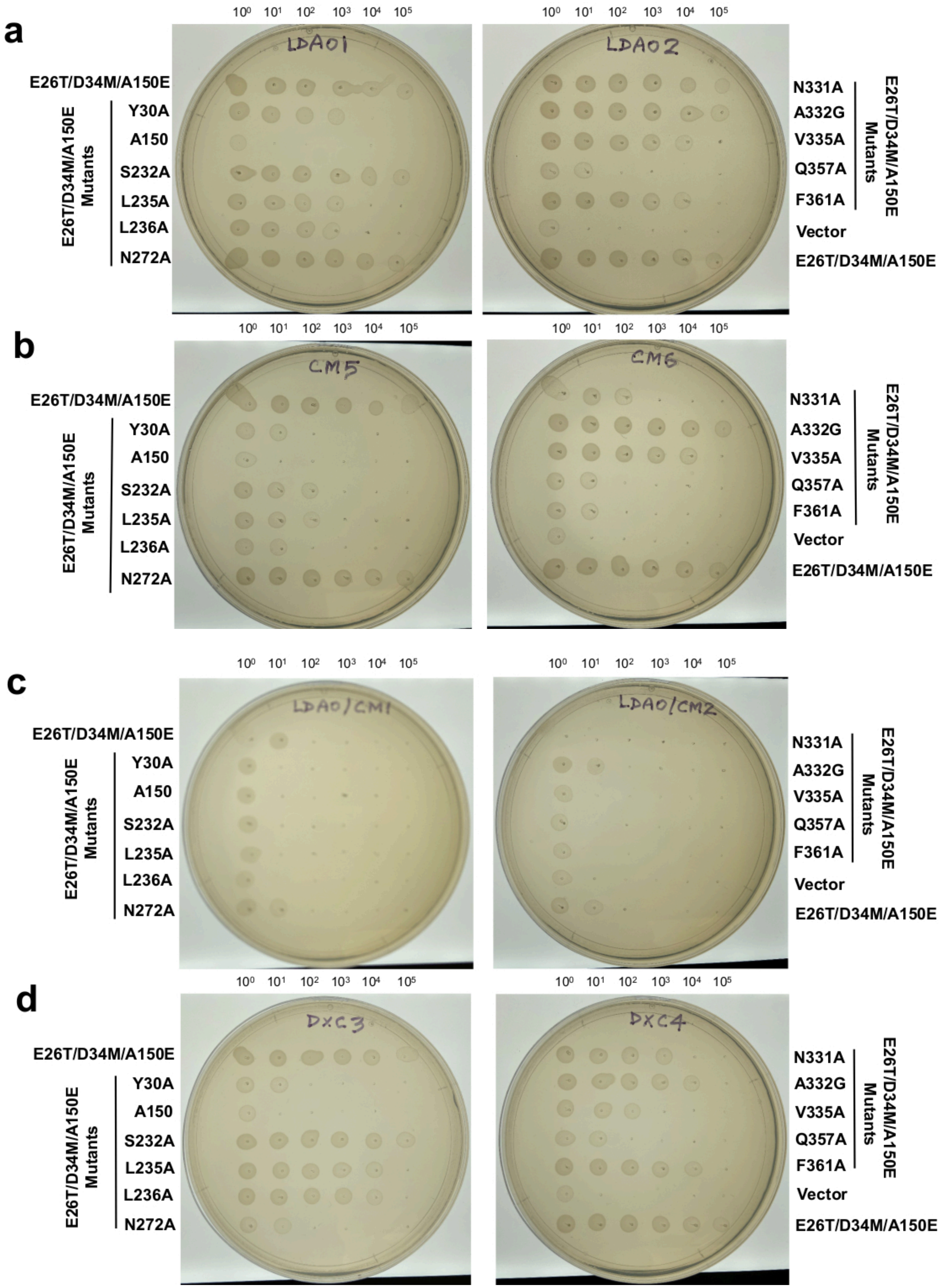
a
b
c
d
10⁰ 10¹ 10² 10³ 10⁴ 10⁵
LDAO1
LDAO2
CM5
CM6
LDAO/CM1
LDAO/CM2
DxC3
DxC4
E26T/D34M/A150E
E26T/D34M/A150E Mutants
Y30A
A150
S232A
L235A
L236A
N272A
N331A
A332G
V335A
Q357A
F361A
Vector

e

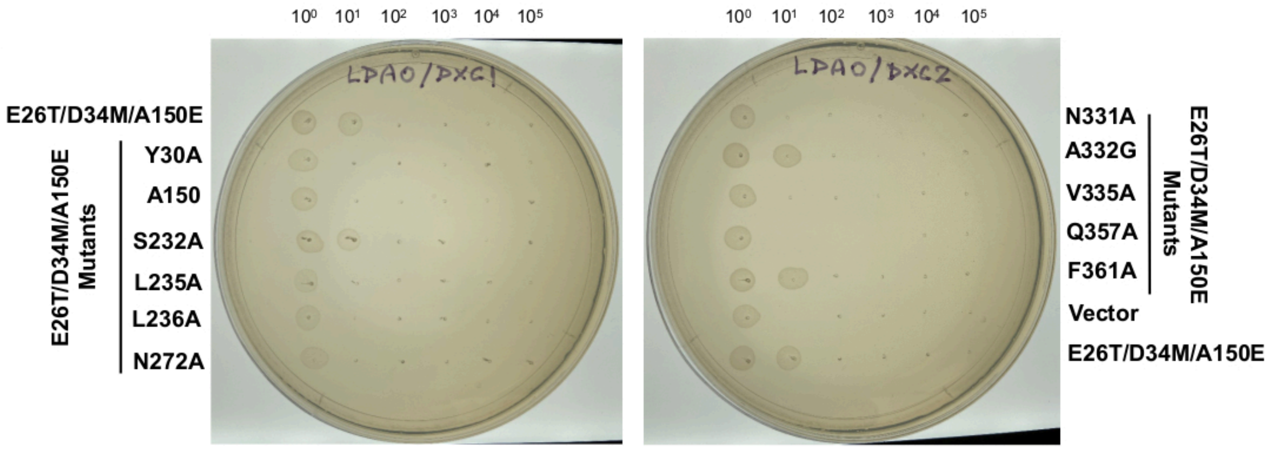

Supplementary Figure 9. Functional characterization of E26T/D34M/A150E variants.

Growth of *E. coli* expressing E26T/D34M/A150E variants on solid media supplemented with kanamycin, IPTG, and LDAO (**a**), chloramphenicol (**b**), both LDAO and chloramphenicol (**c**), deoxycholate (**d**), or both LDAO and deoxycholate (**e**). Five consecutive 10-fold dilutions of bacteria were plated from left to right and incubated overnight. Representative results are shown here in the full-length images of LB-agar plates. Of note, bacterial growth on the control LB-agar plates supplemented with kanamycin and IPTG, i.e., in the absence of cytotoxic drugs, was the same for all the E26T/D34M/A150E variants.

Supplementary Table 1. Data collection and structure refinement statistics

	**Cm-bound E26T/D34M/A150E (low pH)**	**Cm-bound E26T/D34M/A150E (high pH)**	**DXC-bound E26T/D34M/A150E**	**LDAO-bound E26T/D34M/A150E**
Data collection				
Space group	C2	C2	C2	C2
Cell dimensions				
a,b,c (Å)	95.80, 63.43, 109.30	95.05, 65.22,111.46	94.73, 70.41, 112.90	95.01, 66.97, 110.43
α,β,γ (º)	90, 108.85, 90	90, 111.32, 90	90, 112.29, 90	90, 111.10, 90
Resolution (Å)	100.0-2.1 Å	100.0-2.0 Å	100.0-3.0 Å	100.0-3.0 Å
R_{sym}[a]	0.10 (0.72)	0.09 (0.67)	0.12 (0.67)	0.06 (0.57)
$CC_{1/2}$[b]	1.00 (0.42)	1.00 (0.36)	1.00 (0.43)	1.00 (0.35)
I/σ	30.8 (1.1)	31.3 (1.1)	35.8 (1.2)	37.1 (1.2)
Completeness (%)	99.3 (94.4)	95.4 (76.7)	97.1 (90.9)	98.7 (86.4)
Redundancy	10.8 (5.2)	6.6 (3.3)	12.2 (6.1)	4.9 (2.8)
Phasing				
Resolution range	20.0-3.0 Å	20.0-3.0 Å	20.0-3.8 Å	20.0-3.8 Å
Phasing power[c]	1.21	1.01	1.13	1.14
R_{cullis}[d]	0.91	0.97	0.96	0.96
Figure of merit[e]	0.27	0.23	0.24	0.24
Refinement				
Resolution range	15.0-2.1 Å	15.0-2.0 Å	15.0-3.0 Å	15.0-3.0 Å
No. reflections	34557	39352	12474	12518
R_{cryst}[f]/R_{free}[g] (%)	23.6/25.5	22.6/23.3	27.9/29.6	28.4/29.9
No. atoms	3039	3070	2915	2902
$\langle B\rangle_{protein}$	63	52	126	119
$\langle B\rangle_{ligand}$	60	50	118	99
$\langle B\rangle_{water}$	78	75	N.A.	N.A.
r.m.s. deviations				
Bond lengths (Å)	0.007	0.007	0.005	0.006
Bond angles (º)	1.1	1.1	1.2	1.1
Ramachandran				
favored,	100.0%,	100.0%,	98.5%,	99.1%,
allowed,	0%,	0%,	1.5%,	0.9%,
disallowed.	0%.	0%.	0%.	0%.

[a]$R_{sym} = \Sigma| I - \langle I\rangle| / \Sigma I$, where I is the observed intensity of symmetry-related reflections.

[b]$CC_{1/2}$ is the half-split Pearson correlation coefficient.

[c]Phasing power= F_h / E, where F_h is the rms isomorphous/anomalous difference and E the rms residual lack-of-closure.

[d]R_{cullis}(ano)= Σ(||ΔFPH(obs)| - |ΔFPH(calc)||) / Σ|ΔFPH(obs)|, where ΔFPH(obs) and ΔFPH(calc) are the observed and calculated structure factor differences between Bijvoet pairs, respectively.

[e]Figure of merit is defined as weighted mean value of the cosine of phase error.

[f]R_{cryst} = Σ(||F_{obs}| - |F_{calc}||) / Σ(|F_{obs}|), where F_{obs} and F_{calc} are the observed and calculated structure factors, respectively.

[g]R_{free} is the same as R_{cryst} but calculated with 5% of the reflections excluded from structure refinement.

Supplementary Table 2 Distances between the ligands and relevant amino acids

E26T/D34M/A150E	Cm (pH 5)	Cm (pH 8)	DXC	LDAO
**Y30**		3.3 Å		4.4 Å
**A150E**		3.0 Å	2.9 Å	4.3 Å
**S232**		3.0 Å		
**L235**				4.4 Å
**L236**	3.9 Å	3.9 Å		4.3 Å
**N272**			3.0 Å	
**N331**	3.0 Å		3.7 Å	
**A332**			3.8 Å	
**V335**			3.8 Å	
**Q357**	2.8 Å	2.7 Å	2.9 Å	2.8 Å
**F361**	3.8 Å	4.0 Å		
